# On nine species of the spider genus *Eriovixia* (Araneae, Araneidae) from Xishuangbanna, China

**DOI:** 10.3897/zookeys.1034.60411

**Published:** 2021-04-26

**Authors:** Xiaoqi Mi, Shuqiang Li

**Affiliations:** 1 College of Agriculture and Forestry Engineering and Planning, Guizhou Provincial Key Laboratory of Biodiversity Conservation and Utilization in the Fanjing Mountain Region, Tongren University, Tongren 554300, Guizhou, China Tongren University Tongren China; 2 Institute of Zoology, Chinese Academy of Sciences, Beijing 100101, China Institute of Zoology, Chinese Academy of sciences Beijing China

**Keywords:** Misidentified, morphology, new species, orb-weaver spider, taxonomy

## Abstract

Species of the genus *Eriovixia* Archer, 1951 from Menglun Town, Xishuangbanna, Yunnan, China are reviewed, including seven new species: *E. ganae***sp. nov.** (♂♀), *E. liuhongi***sp. nov.** (♂♀), *E. tangi***sp. nov.** (♂♀), *E. wangchengi***sp. nov.** (♂♀), *E. yaoi***sp. nov.** (♂♀), *E. yinae***sp. nov.** (♂♀) and *E. zhengi***sp. nov.** (♂♀). The male of *E. yunnanensis* (Yin, Wang, Xie & Peng, 1990) is described for the first time. The previous description of *E. yunnanensis* from Tengchong, Yunnan by Mi et al. (2010) refers to *E. pengi***sp. nov.** (♂♀). Diagnostic photos of the habitus and copulatory organs of the new species and *E. yunnanensis* from Xishuangbanna are provided.

## Introduction

*Eriovixia* Archer, 1951 is a genus of the spider family Araneidae Clerck, 1757, with 25 named species occurring in Africa (three species) and Asia (22). All of the African species were originally described more than 100 years ago and there have been no publications on them in the last 50 years. In Japan, the species have been well studied by Tanikawa (1999, 2007, 2009) and species from China, India and Bangladesh have been considered by Han and Zhu (2010), Mi et al. (2010), Ahmed et al. (2016), Zhou et al. (2017), Biswas and Raychaudhuri (2018) and Basumatary et al. (2019). To date, China has the highest diversity, with 15 species, mainly distributed in southwest China (WSC 2020); the northernmost species of the genus in China is *E. cavaleriei* (Schenkel, 1963) found in Beijing (Li and Lin 2016).

Xishuangbanna Tropical Botanical Garden and surrounding areas in Menglun Town lie in Yunnan Province, southwest China. Seven hundred and eighty two species of spiders have been recorded from this area through an “All Species Inventory” over the past 15 years (Li 2020). While examining specimens from Xishuangbanna Tropical Botanical Garden and surrounding areas, 13 *Eriovixia* species were identified, including six named species: *E. bannaensis* Zhou, Zhu & Zhang, 2017, *E. excelsa* (Simon, 1889), *E. menglunensis* (Yin, Wang, Xie & Peng, 1990), *E. nigrimaculata* Han & Zhu, 2010, *E. poonaensis* (Tikader & Bal, 1981) and *E. yunnanensis* (Yin, Wang, Xie & Peng, 1990) and seven new species. The goal of this paper is to describe the new species and re-describe *E. yunnanensis*. *E. yunnanensis* from Tengchong reported by Mi et al. (2010) was a misidentification and their description actually refers to *E. pengi* sp. nov.

## Material and Methods

All specimens were collected by beating shrubs, fogging or hand collecting and kept in 75% ethanol. Most of the specimens were collected in Xishuangbanna and are deposited in the Institute of Zoology, Chinese Academy of Sciences (IZCAS); comparative specimens of *E. cavaleriei* (Schenkel, 1963) and *E. jianfengensis* Han & Zhu, 2010 were collected in Guizhou and Hainan, respectively and are deposited in Tongren University (TRU); types of *E. pengi* sp. nov. are deposited in Hunan Normal University (HNU) and the California Academy of Sciences (CAS). The specimens were examined with an Olympus SZ51 stereomicroscope. The epigyna were cleared in trypsin enzyme solution for examination and imaging. Left male palps were dissected in ethanol for examination, description and imaging. Photos of the habitus and copulatory organs were taken with a Kuy Nice CCD mounted on an Olympus BX51 compound microscope. Compound focus images were generated using Helicon Focus v. 6.7.1.

All measurements are given in millimetres (mm). Leg measurements are given as: total length (femur, patella + tibia, metatarsus, tarsus). References to figures in the cited papers are listed in lowercase (fig. or figs); figures in this paper are noted with an initial capital (Fig. or Figs). Abbreviations used in the figures are as follows: C conductor; CD copulatory duct; CO copulatory opening; E embolus; K keel; MA median apophysis; MP median plate; S spermathecae; TA terminal apophysis.

Depository acronyms: **CAS**California Academy of Sciences; **HNU**Hunan Normal University; **IZCAS**Institute of Zoology, Chinese Academy of Sciences, **TRU** Tongren University.

## Taxonomy

**Family Araneidae Clerck, 1757**

**Subfamily Araneinae Clerck, 1757**

### Genus Eriovixia

Taxon classificationAnimaliaAraneaeAraneidae

Archer, 1951

751D4CC2-5E43-5612-8E78-A4C392571F07

Eriovixia Archer, 1951a: 18.
Simonarachne Archer, 1951b: 28.
Heurodes Yaginuma & Archer, 1959: 35.
Eriovixia : Grasshoff, 1986: 4; Yin et al., 1997: 294; Tanikawa, 1999: 42; Tanikawa, 2007: 90; Han and Zhu 2010: 2610.
Tukaraneus Barrion & Litsinger, 1995: 644.

#### Type species.

*Araneus rhinura* Pocock, 1900 from Benito River in Equatorial Guinea.

#### Description.

Small to medium-sized. Carapace pear-shaped, covered with setae, fovea depressed, male often with a cephalic protuberance below AMEs. Chelicerae yellow, with 4 promarginal and 3 retromarginal teeth (with 2 or 4 retromarginal teeth in some species). Endites and labium often dark at base and paler distally. Leg I longest, leg III shortest, Leg II longer than leg IV, femur II of males with a groove at base, femur II, patella II and tibia II of males with 16–23 macrosetae. Abdomen longer than wide (slightly wider than long in *E. sakiedaorum* Tanikawa, 1999), blunt anteriorly and tapered posteriorly, extending more or less beyond the spinnerets. Male palp lacking long patellar bristles; cymbium longer than wide; paracymbium finger-like; median apophysis prominent, often with dorsal spur(s); conductor wide and thick; embolus short; terminal apophysis varying according to species. Epigyne strongly sclerotised, with a posteriorly directed, rimmed scape; copulatory openings situated posteriorly; spermathecae round, ovoid or kidney-shaped.

#### Diagnosis.

The genus is similar to some *Neoscona* in epigyne structure, but can be distinguished by: 1) small to medium size (♀♀3.00–9.80, ♂♂ 2.50–7.20) vs. medium to large size (♀♀ 4.50–15.00, ♂♂ 3.75–10.80); 2) the female spinnerets situated on the posterior 1/2 to 1/3 of abdomen vs. close to the posterior edge of abdomen; 3) lacking long patellar bristles vs. with 2 long bristles; 4) coxae I in male without apophysis vs. with a hook, like an apophysis; and 5) males often having a cephalic protuberance below AMEs vs. absent.

#### Composition and distribution.

A total of 25 described species with three in Africa and 22 in Asia.

#### Comments.

The differences between the males of the type species and Asian species are unknown because the palp of the type species was not well illustrated or photographed and the Asian species may belong to a separate genus.

### Eriovixia ganae sp. nov.

Taxon classificationAnimaliaAraneaeAraneidae

CD0B67B0-D5D4-58F8-A2A8-78DA82013E7E

http://zoobank.org/F7E6F4F5-73DB-45CA-9BB4-D38FB468C066

**Figure 1.**
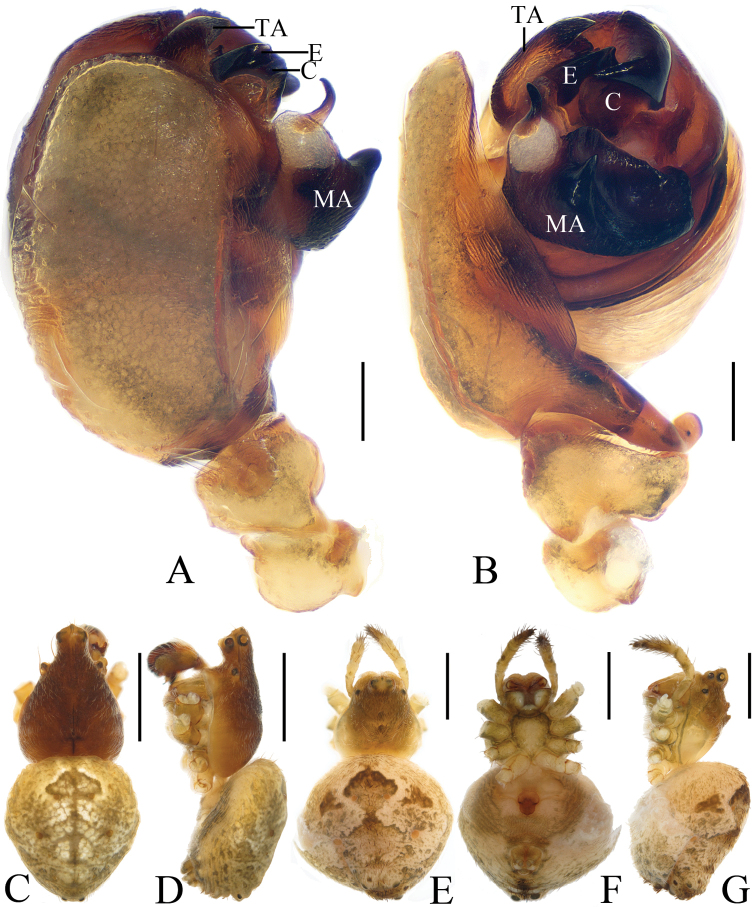
*Eriovixia ganae* sp. nov., male holotype and female paratype **A** male palp, dorsal view **B** ibid., prolateral view **C** holotype habitus, dorsal view **D** ibid., lateral view **E** paratype habitus, dorsal view **F** ibid., ventral view **G** ibid., lateral view. Scale bars: 0.1 mm (**A, B**); 1 mm (**C–G**).

**Figure 2.**
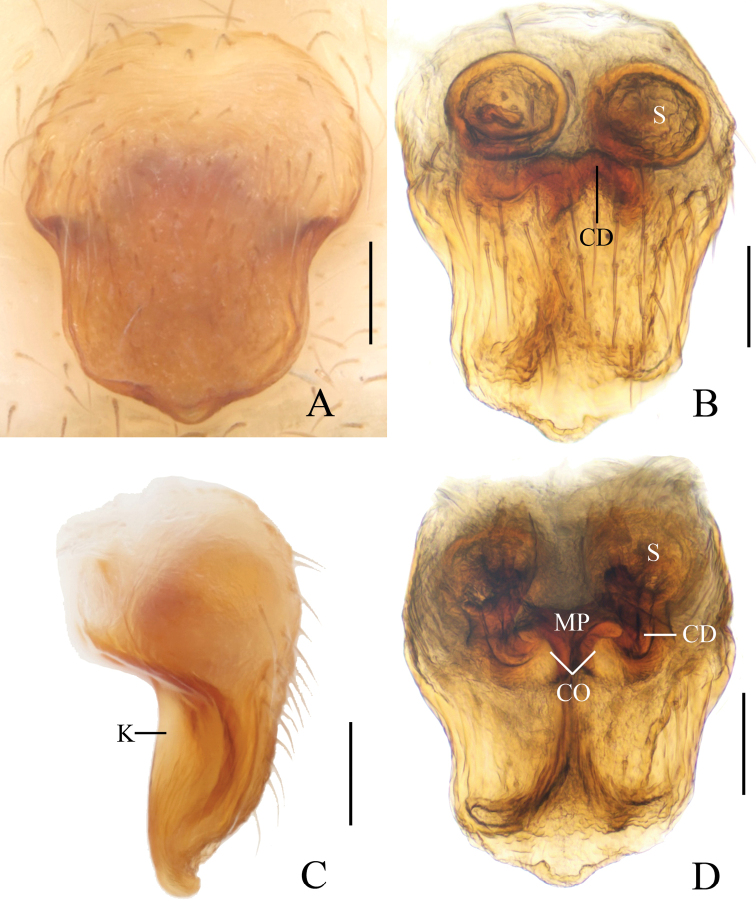
*Eriovixia ganae* sp. nov., female paratype, epigyne **A** ventral view **B** ventral view **C** lateral view **D** dorsal view. Scale bars: 0.1 mm.

Figs 1 , 2 , 17A , 19A , 20A

#### Type material.

Holotype ♂ (IZCAS-Ar41648), China: Yunnan, Xishuangbanna, Mengla County, Menglun Township, Menglun Nature Reserve, *Magnolia baillonii* plantation (about 20 yr) (21°53.82'N, 101°17.07'E, 613 m alt.), 18.VIII.2007 p.m., G. Zheng leg. Paratypes: 1♂1♀ (IZCAS-Ar41649–40650), rubber plantation (about 20 yr) (21°54.50'N, 101°16.33'E, 586 m alt.), 17.VII.2007, G. Zheng leg.; 1♂ (IZCAS-Ar41651), *Magnolia baillonii* plantation (about 20 yr.) (21°54.77'N, 101°16.04'E, 556 m alt.), 18.VII.2007. G. Zheng leg.; 1♀ (IZCAS-Ar41652), Yulinjiegou Scenic Spot (21°55.20'N, 101°16.20'E, 553 m alt.), 2.VIII.2018 night, C. Wang et al. leg.; 1♀ (IZCAS-Ar41653), Baihuayuan Scenic Spot (21°55.60'N, 101°14.87'E, 541 m alt.), 4.VIII.2018 night, C. Wang et al. leg.; 1♂ (IZCAS-Ar41654), palm plantation (21°55.47'N, 101°15.05'E, 554 m alt.), 13.VIII.2018, C. Wang et al. leg.; 1♀ (IZCAS-Ar41655), Baizhuyuan Scenic Spot (21°55.83'N, 101°14.93'E, 542 m alt.), 30.IV.2019, C. Wang leg. Other material examined: 4♀(IZCAS-Ar41656), Qihuayihui Scenic Spot (21°55.61'N, 101°14.94'E, 603 m alt.), 2.V.2019 night, C. Wang leg.; 2♂1♀ (IZCAS-Ar41657), same locality (21°55.61'N, 101°14.94'E, 603 m alt.), 13.V.2019, C. Wang leg.; 1♀ (IZCAS-Ar41658), #2 site in Mafengzhai Village (21°53.59'N, 101°17.30'E, 546 m alt.), 4.V.2019, Y. Tong et al. leg.; 1♀ (IZCAS-Ar41659), riverside near Baizhuyuan Scenic Spot (21°55.83'N, 101°14.93'E, 542 m alt.), 9.V.2019, C. Wang leg.

#### Etymology.

The specific name is a patronym of Mrs. Jiahui Gan (Tongren University), one of the collectors of the type specimens; noun (name) in genitive case.

#### Diagnosis.

The new species resembles *E. nigrimaculata* in habitus, but can be distinguished from the latter by the: 1) female carapace with 2 tubercles vs. lacking tubercles (Han and Zhu 2010: fig. 3C); 2) embolus wide and bent distally in prolateral view vs. thin and not bent (Han and Zhu 2010: fig. 11D); 3) median apophysis with 2 dorsal spurs vs. 3 spurs (Han and Zhu 2010: figs 11D and E); 4) scape nearly square vs. long and triangular (Han and Zhu 2010: figs 11A–C); and 5) median plate keeled vs. not keeled (Han and Zhu 2010: fig. 11C).

#### Description.

***Male*** (holotype, Figs 1A–D, 17A, 19A, 20A). Total length 3.10. Carapace 1.60 long, 1.20 wide. Abdomen 1.60 long, 1.60 wide. Clypeus 0.25 high. Eye sizes and interdistances: AME 0.08, ALE 0.05, PME 0.08, PLE 0.05, AME–AME 0.13, AME–ALE 0.25, PME–PME 0.23, PME–PLE 0.38, MOA length 0.28 with anterior width 0.28 and posterior width 0.35. Leg measurements: I 5.15 (1.55, 1.85, 1.20, 0.55), II 3.95 (1.35, 1.30, 0.85, 0.45), III 2.50 (0.90, 0.85, 0.45, 0.30), IV 3.40 (1.10, 1.15, 0.75, 0.40). Carapace dark brown, cervical groove inconspicuous, cephalic protuberance shorter than AME diameter. Chelicerae yellow with 4 promarginal teeth and 2 retromarginal teeth. Sternum yellow, with irregular dark patches and a large white spot posteriorly, bearing sparse darker setae. Legs yellowish-brown with inconspicuous rings. Abdomen as long as wide, dorsum greyish-yellow with a rhombic dark patch, terminus with two pairs of very short tubercles; ventre greyish-yellow, with a pair of white spots and brown patches. Spinnerets yellowish-brown.

*Palp* (Figs 1A, B and 17A): median apophysis prominent, about 3/5 width of the bulb diameter in apical view, with 2 spurs basally and concave distally; embolus long, flattened and bent distally, curled; terminal apophysis triangular in apical view, about 1.35 times longer than wide, tapered distally.

***Female*** (paratype IZCAS-Ar40650, Figs 1E–G, 2). Total length 3.40. Carapace 1.50 long, 1.30 wide. Abdomen 2.10 long, 2.20 wide. Clypeus 0.15 high. Eye sizes and interdistances: AME 0.08, ALE 0.05, PME 0.08, PLE 0.05, AME–AME 0.15, AME–ALE 0.20, PME–PME 0.28, PME–PLE 0.30, MOA length 0.25 with anterior width 0.28 and posterior width 0.35. Leg measurements: I 5.45 (1.85, 1.95, 1.15, 0.50), II 4.20 (1.45, 1.50, 0.80, 0.45), III 2.55 (0.80, 0.90, 0.45, 0.40), IV 3.95 (1.35, 1.35, 0.80, 0.45). Habitus similar to that of male, but carapace with 2 short tubercles.

*Epigyne* (Fig. 2) square shaped, about 1.2 times longer than wide; median plate keeled; copulatory openings round; copulatory ducts twisted and very short, about as long as 2 diameters of spermathecae; spermathecae globular, separated from each other.

#### Variation.

Total length: ♂♂ 2.50–3.10; ♀♀ 3.20–4.00.

#### Distribution.

China (Yunnan).

### Eriovixia liuhongi sp. nov.

Taxon classificationAnimaliaAraneaeAraneidae

3397350D-3311-57BD-AF1C-63BD1C9C0C93

http://zoobank.org/F89608A2-7C90-4B38-99E8-E149AC104680

**Figure 3.**
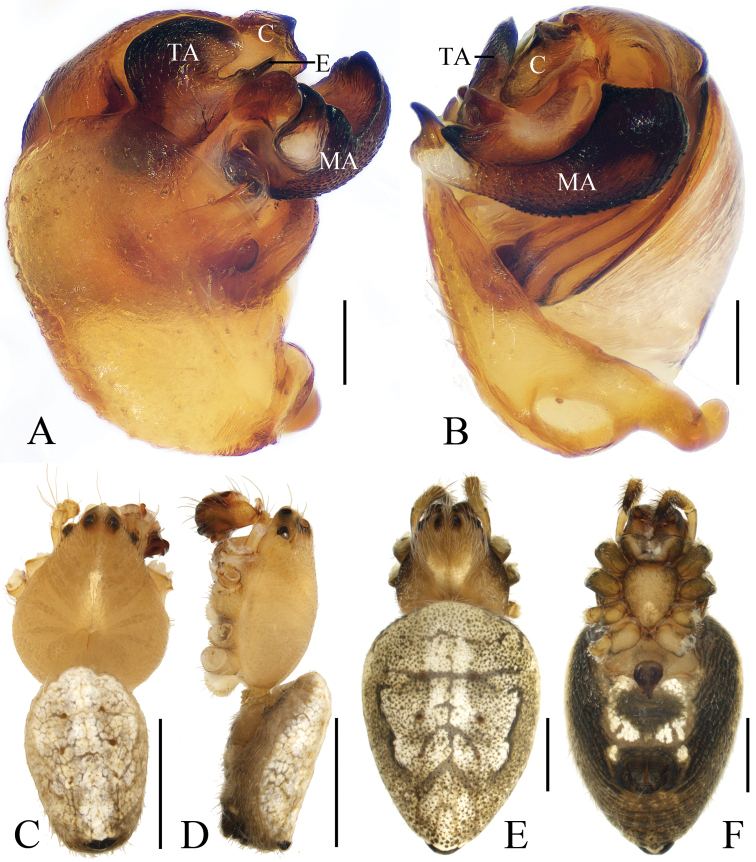
*Eriovixia liuhongi* sp. nov., male holotype and female paratype **A** male palp, dorsal view **B** ibid., prolateral view **C** holotype habitus, dorsal view **D** ibid., lateral view **E** paratype habitus, dorsal view **F** ibid., ventral view. Scale bars: 0.1 mm (**A, B**); 1 mm (**C–F**).

**Figure 4.**
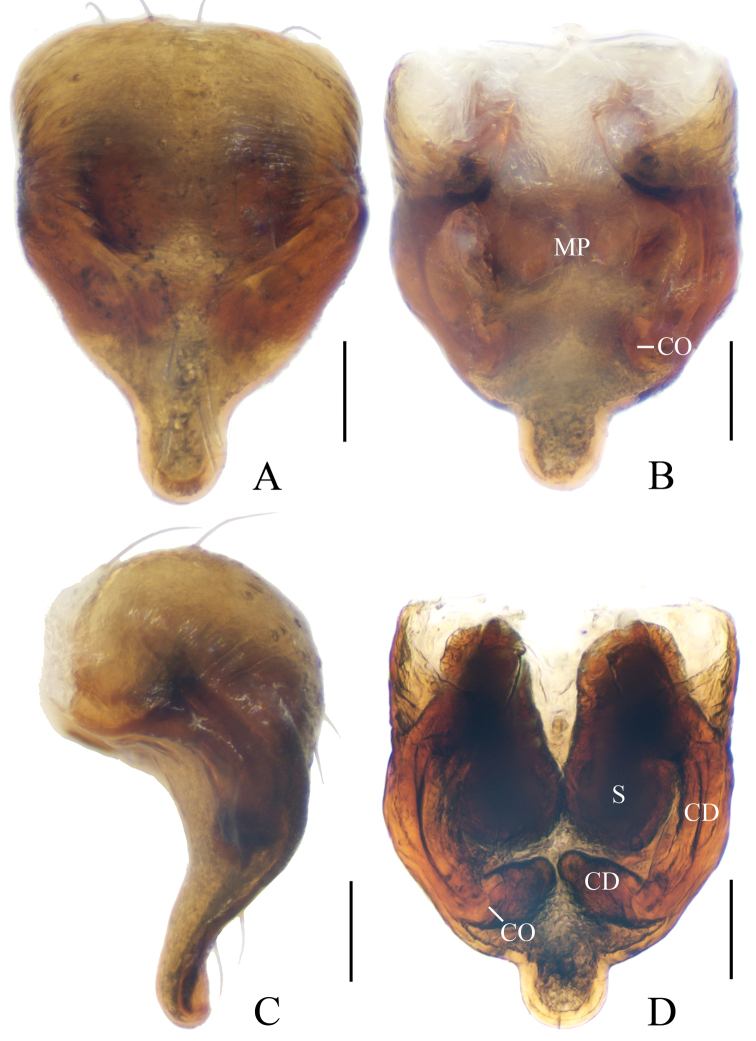
*Eriovixia liuhongi* sp. nov., female paratype, epigyne. A. ventral view **B** dorsal view **C** lateral view **D** dorsal view. Scale bars: 0.1 mm.

Figs 3 , 4 , 17B , 19B , 20B

#### Type material.

Holotype ♂ (IZCAS-Ar41660), China: Yunnan, Xishuangbanna, Mengla County, Menglun Township, Menglun Nature Reserve, Xishuangbanna Tropical Botanical Garden, eastern part (21°54.07'N, 101°16.36'E, 544 m alt.), 16.VII.2018, X. Mi et al. leg. Paratypes: 2♀ (IZCAS-Ar41661–41662), same locality (21°54.07'N, 101°16.36'E, 544 m alt.), 22.VII.2018, X. Mi et al. leg.

#### Comparative material.

*Eriovixia cavaleriei* (Schenkel, 1963), 7♂4♀ (TRU), CHINA: Guizhou, Tongren, Bijiang District, Chuandong Township, Jianyan Village (27°50.85'N, 109°14.26'E, 480 m alt.), 27.IV.2019, X. Mi et al. leg.

#### Etymology.

The specific name is the full name of Mr. Hong Liu (Tongren, China), one of the collectors of the type specimens; noun (name) in genitive case.

#### Diagnosis.

The female of *E. liuhongi* sp. nov. resembles those of *E. kachugaonensis*Basumatary et al., 2019 in general appearance, but can be distinguished from the latter by the: 1) spinnerets situated at posterior 1/3 of the abdomen vs. posterior 1/2 (Basumatary et al. 2019: figs 5–6); 2) spermathecae kidney shaped vs. globular (Basumatary et al. 2019: figs 10–13); 3) spermathecae touching each other vs. separated (Basumatary et al. 2019: figs 10–13); and 4) copulatory ducts extend to the lateral edges of the epigyne vs. extended to the central part of the epigyne (Basumatary et al. 2019: figs 10–13). The male and female resemble those of *E. cavaleriei* (Schenkel, 1963) by the shape of the copulatory organs, but can be distinguished by: 1) lacking a white stripe on the anterior edge of abdomen vs. 2 white stripes extending to the anterior edge (Schenkel 1963: fig. 95a); 2) outline of the folium is wavy vs. deeply toothed (Schenkel 1963: fig. 95a); 3) terminal apophysis fused with embolus at base vs. conspicuously separated; and 4) spermathecae kidney shaped vs. ovoid.

#### Description.

***Male*** (Holotype, Figs 3A–D, 17B, 19B). Total length 2.70. Carapace 1.50 long, 1.30 wide. Abdomen 1.30 long, 1.00 wide. Clypeus 0.10 high. Eye sizes and interdistances: AME 0.08, ALE 0.05, PME 0.08, PLE 0.05, AME–AME 0.10, AME–ALE 0.15, PME–PME 0.10, PME–PLE 0.20, MOA length 0.28 with anterior width 0.28 and posterior width 0.28. Leg measurements: I 4.90 (1.60, 1.75, 1.10, 0.45), II 3.85 (1.25, 1.30, 0.90, 0.40), III 2.20 (0.75, 0.70, 0.45, 0.30), IV 3.60 (1.00, 1.15, 1.10, 0.35). Carapace yellow, cervical groove inconspicuous, lacking cephalic protuberance. Chelicerae and endites yellow, labium greyish-yellow, sternum yellow, with irregular dark patches and sparse darker setae. Legs yellow with dark rings. Abdomen oval, yellowish-white, dorsum with a large, pale folium and a large black spot posteriorly; ventre greyish-yellow, with pair of arcuate white patches and pair of white spots anterior to brown spinnerets.

*Palp* (Figs 3A, B, 17B): median apophysis as wide as the bulb diameter, with 2 spurs prolaterally, retrolateral end curled; embolus straight, about half length of the terminal apophysis in prolateral view; terminal apophysis almost semicircular in prolateral view and fused with embolus at base; conductor flat, with a small tip in apical view.

***Female*** (paratype IZCAS-Ar41661, Figs 3E, F, 4, 20B). Total length 4.80. Carapace 1.80 long, 1.30 wide. Abdomen 3.50 long, 2.40 wide. Clypeus 0.08 high. Eye sizes and interdistances: AME 0.10, ALE 0.08, PME 0.10, PLE 0.05, AME–AME 0.13, AME–ALE 0.18, PME–PME 0.13, PME–PLE 0.23, MOA length 0.28 with anterior width 0.28 and posterior width 0.28. Leg measurements: I 5.05 (1.60, 1.85, 1.15, 0.45), II 4.50 (1.35, 1.55, 1.15, 0.45), III 2.55 (0.85, 0.85, 0.50, 0.35), IV 4.10 (1.30, 1.45, 0.95, 0.40). Habitus similar to that of male but the outline of the dorsal abdominal folium more obvious.

*Epigyne* (Fig. 4) about 1.4 times longer than wide; scape triangular, narrowed near tip, copulatory openings arcuate; copulatory ducts very long, directed towards lateral margin; spermathecae long, kidney shaped, touching each other.

#### Variation.

Total length: ♀♀ 4.20–4.80.

#### Distribution.

China (Yunnan).

### Eriovixia tangi sp. nov.

Taxon classificationAnimaliaAraneaeAraneidae

53F111CC-1AEC-5445-B817-E45DB49C7F6A

http://zoobank.org/80703645-2A7E-49D2-81EF-E3D6F613F917

**Figure 5.**
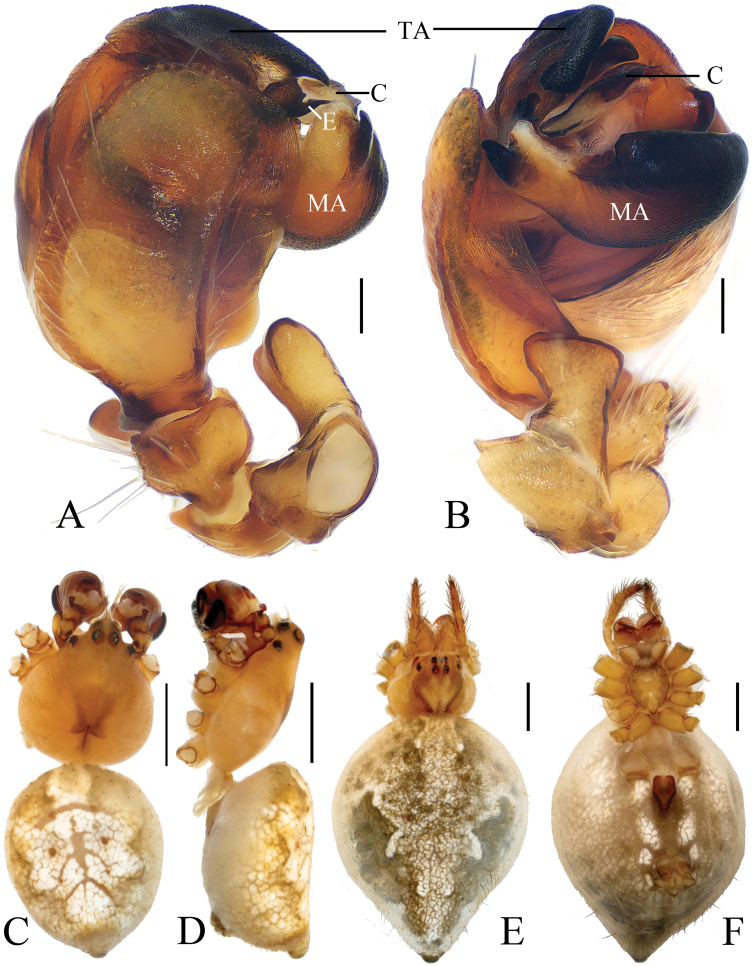
*Eriovixia tangi* sp. nov., male holotype and female paratype **A** male palp, dorsal view **B** ibid., prolateral view **C** holotype habitus, dorsal view **D** ibid., lateral view **E** paratype habitus, dorsal view **F** ibid., ventral view. Scale bars: 0.1 mm (**A, B**); 1 mm (**C–F**).

**Figure 6.**
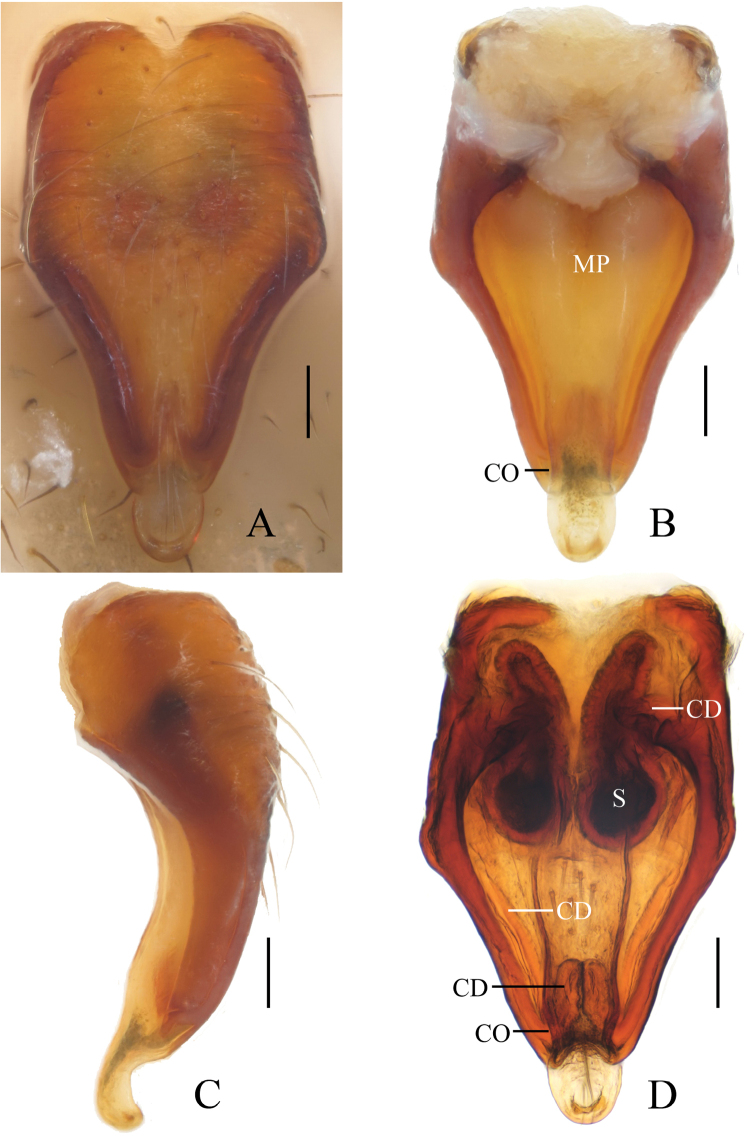
*Eriovixia tangi* sp. nov., female paratype, epigyne **A** ventral view **B** dorsal view **C** lateral view **D** dorsal view. Scale bars: 0.1 mm.

Figs 5 , 6 , 17C , 19C , 20C

#### Type material.

Holotype ♂ (IZCAS-Ar41663), China: Yunnan, Xishuangbanna, Mengla County, Menglun Township, Menglun Nature Reserve, valley tropical seasonal rainforest (21°54.97'N, 101°16.43'E, 551 m alt.), 1.XII.2009, G. Tang et al. leg. Paratypes: 1♂ (IZCAS-Ar41664), same data as holotype; 1♀ (IZCAS-Ar41665), rubber plantation (about 20 yr) (21°54.50'N, 101°16.33'E, 586 m alt.), 17.VII.2007 a.m. G. Zheng leg.; 1♂ (IZCAS-Ar41666), Lvshilin Forest Park (21°54.71'N, 101°16.90'E, 664 m alt.), 15.XI.2009, G. Tang et al. leg.; 1♀ (IZCAS-Ar41667), Teak plantation (21°54.12'N, 101°16.17'E, 549 m), 8.VIII.2018, Z. Bai et al. leg.; 1♀ (IZCAS-Ar41668), Baihuayuan Scenic Spot (21°55.60'N, 101°14.87'E, 541 m alt.), 25.IV.2019 night, C. Wang leg.; 1♀ (IZCAS-Ar41669), #1 site in Mafengzhai Village (21°53.45'N, 101°17.40'E, 543 m alt.), 29.IV.2019, Y. Tong et al. leg.; 1♂ (IZCAS-Ar41670), G213 roadside (21°53.75'N, 101°17.08'E, 619 m alt.), 1.V.2019, Y. Tong et al. leg. Other material examined: 1♂ (IZCAS-Ar41671), Qihuayihui Scenic Spot (21°55.61'N, 101°14.94'E, 603 m alt.), 2.V.2019 night, C. Wang leg.; 1♂ (IZCAS-Ar41672), same locality (21°55.61'N, 101°14.94'E, 603 m alt.), 14.V.2019 night, C. Wang leg; 1♂ (IZCAS-Ar41673), #2 site in Mafengzhai Village (21°53.59'N, 101°17.30'E, 546 m alt.), 4.V.2019, Y. Tong et al. leg.; 1♀ (IZCAS-Ar41674), #3 site in Mafengzhai Village (21°53.68'N, 101°17.33'E, 539 m alt.), 8.V.2019, Y. Tong et al. leg.; 2♀ (IZCAS-Ar41675), Lvshilin Forest Park (21°53.843'N, 101°16.84'E, 550 m alt.), 10.V.2019, Z. Bai et al. leg.

#### Etymology.

The specific name is a patronym in honour of the late Dr. Guo Tang for his contribution to the taxonomy of the spider family Thomisidae in China and he was one of the collectors of the type specimens; noun (name) in genitive case.

#### Diagnosis.

*Eriovixia tangi* sp. nov. resembles *E. cavaleriei* by having a similar abdominal pattern, but it can be distinguished from the latter by the following characters: 1) male palpal femur with large apophysis vs. absent; 2) kidney-shaped, long spermathecae vs. ovoid; and 3) terminal apophysis covered with small denticles vs. lacking denticles.

#### Description.

***Male*** (holotype, Figs 5A–D, 17C, 19C, 20C). Total length 4.10. Carapace 1.60 long, 1.60 wide. Abdomen 2.40 long, 1.80 wide. Clypeus 0.10 high. Eye sizes and interdistances: AME 0.10, ALE 0.08, PME 0.10, PLE 0.08, AME–AME 0.15, AME–ALE 0.23, PME–PME 0.15, PME–PLE 0.30, MOA length 0.33 with anterior width 0.33 and posterior width 0.30. Leg measurements: I 7.70 (2.50, 2.80, 1.75, 0.65), II 5.40 (1.75, 1.75, 1.30, 0.60), III 3.45 (1.20, 1.15, 0.65, 0.45), IV 4.85 (1.50, 1.65, 1.20, 0.50). Carapace yellow, darkened around fovea, with 2 long setae anteriorly, cervical groove inconspicuous, cephalic protuberance below wider than an AME diameter. Chelicerae, endites, labium and sternum yellow. Legs yellow, without rings. Abdomen about 1.2 times longer than wide, dorsum with a large, pale folium and a dark spot posteriorly; ventre greyish-yellow with a pair of white arcuate patches and 2 pairs of white spots around spinnerets.

*Palp* (Figs 5A, B, 17C): with a large, distally expanded femoral apophysis; median apophysis slightly wider than bulb diameter in apical view, curled laterally; embolus small, square at base in prolateral view, covered with small denticles; terminal apophysis large, rounded, well sclerotised with dozens of fine denticles; conductor flat, about 3/5 width of bulb diameter in apical view.

***Female*** (paratype IZCAS-Ar41665, Figs 5E, F, 6). Total length 6.50. Carapace 2.00 long, 1.60 wide. Abdomen 4.60 long, 3.30 wide. Clypeus 0.08 high. Eye sizes and interdistances: AME 0.13, ALE 0.10, PME 0.13, PLE 0.10, AME–AME 0.13, AME–ALE 0.23, PME–PME 0.15, PME–PLE 0.30, MOA length 0.30 with anterior width 0.30 and posterior width 0.30. Leg measurements: I 7.50 (2.50, 2.80, 1.55, 0.65), II 5.80 (1.95, 2.15, 1.10, 0.60), III 3.40 (1.15, 1.10, 0.70, 0.45), IV 4.95 (1.70, 1.80, 0.95, 0.50). Habitus similar to that of male, but cervical groove more conspicuous, cephalic region darker, sternum with a large pale spot posteriorly and abdomen droplet-shaped and more pointed posteriorly.

*Epigyne* (Fig. 6) about 1.8 times longer than wide, triangular and tapering posteriorly, depressed anteriorly; copulatory openings close to tip of scape; copulatory ducts very long, touching each other at their origin, then turned dorsally and twisted near lateral edge, connected to spermathecae medially; spermathecae long, kidney shaped, posterior end larger than anterior end, touching each other posteriorly.

#### Variation.

Total length: ♂♂ 3.60–4.10; ♀♀ 4.60–6.50.

#### Distribution.

China (Yunnan).

### Eriovixia wangchengi sp. nov.

Taxon classificationAnimaliaAraneaeAraneidae

A45DAF3D-C7E4-58B8-9168-D6F2A44309D3

http://zoobank.org/9206572C-4D2C-4DC6-89C2-02C88D85594C

**Figure 7.**
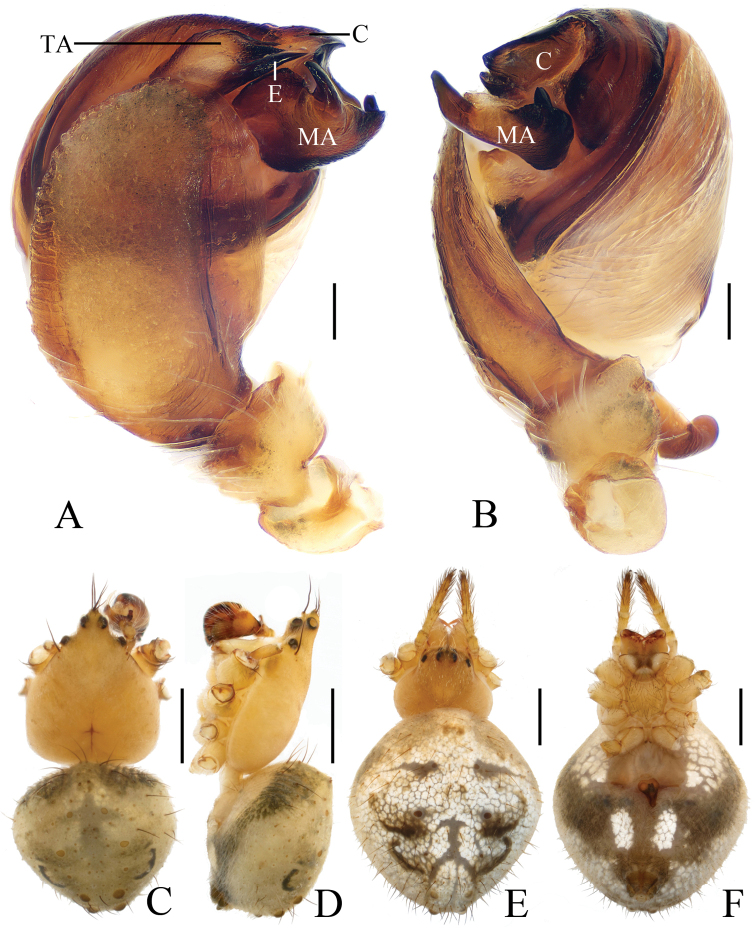
*Eriovixia wangchengi* sp. nov., male holotype and female paratype **A** male palp, dorsal view **B** ibid., prolateral view **C** holotype habitus, dorsal view **D** ibid., lateral view **E** paratype habitus, dorsal view **F** ibid., ventral view. Scale bars: 0.1 mm (**A, B**); 1 mm (**C–F**).

**Figure 8.**
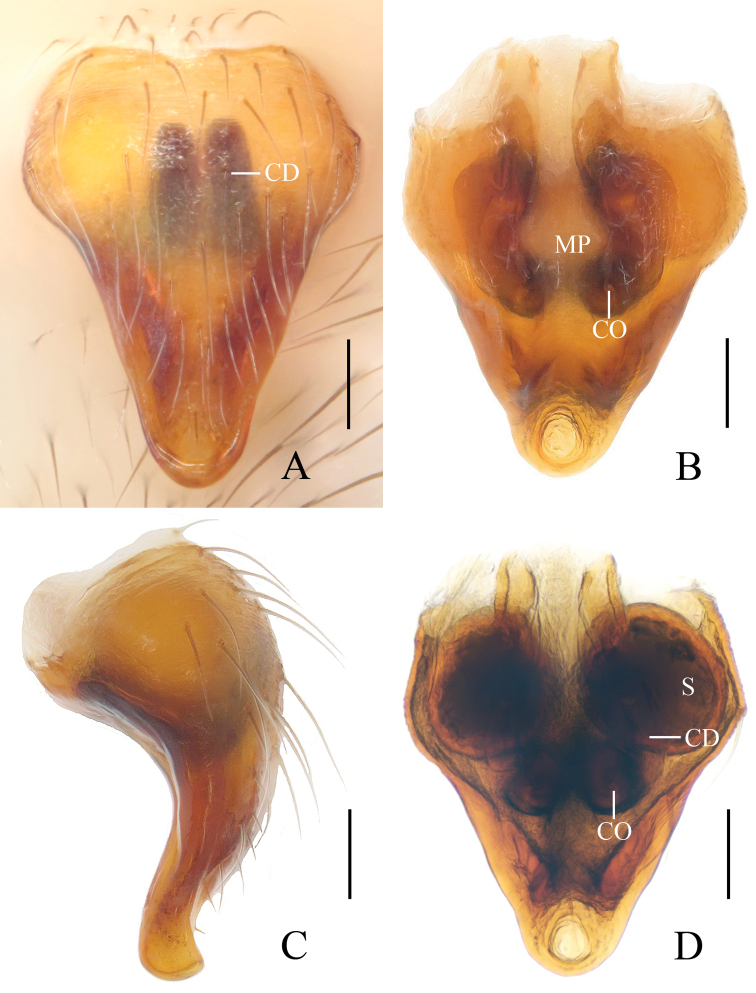
*Eriovixia wangchengi* sp. nov., female paratype, epigyne **A** ventral view **B** dorsal view **C** lateral view **D** dorsal view. Scale bars: 0.1 mm.

Figs 7 , 8 , 17D , 19D , 20D

#### Type material.

Holotype ♂ (IZCAS-Ar41676), China: Yunnan, Xishuangbanna, Mengla County, Menglun Township, Menglun Nature Reserve, *Anogeissus acuminata* plantation (about 20 yr.) (21°53.99'N, 101°16.81'E, 611 m alt.), 19.VIII.2007 G. Zheng leg. Paratypes: 1♀ (IZCAS-Ar41677), G213 roadside (21°54.46'N, 101°16.76'E, 644 m alt.), 20.XI.2009, G. Tang et al. leg.; 1♀ (IZCAS-Ar41678), secondary tropical forest near Lvshilin Forest Park (21°54.38'N, 101°16.82'E, 627 m alt.), 23.XI.2009, G. Tang et al. leg.; 1♀ (IZCAS-Ar41679), G213 roadside (21°54.28'N, 101°16.75'E, 629 m alt.), 25.IV.2019, Z. Bai et al. leg.; 1♀ (IZCAS-Ar41680), Masuoxing Village (21°54.02'N, 101°16.90'E, 561 m alt.), 27.IV.2019, Y. Tong et al. leg.; 1♀ (IZCAS-Ar41681), G213 roadside (21°54.34'N, 101°16.79'E, 618 m alt.), 2.V.2019, Y. Tong et al. leg.; 1♂ (IZCAS-Ar41682), G213 roadside ((21°54.05'N, 101°16.93'E, 597 m alt.), 9.V.2019, Y. Tong et al. leg. Other material examined: 6♂2♀ (IZCAS-Ar41683), G213 roadside (21°52.65'N, 101°16.27'E, 575 m alt.), 31.VII.2018, Z. Bai et al. leg.

#### Comparative material.

*Eriovixia jianfengensis* Han & Zhu, 2010, 1♂ (TRU), CHINA: Hainan, Ledong County, Jianfeng Township, Jianfengling National Natural Reserve (18°44.45'N, 108°51.49'E, 856 m alt.), 11.IV.2019, C. Wang et al. leg.; 2♀ (TRU), same locality (18°44.61'N, 108°51.24'E, 812 m alt.), 12.IV.2019, C. Wang et al. leg.

#### Etymology.

The specific name is the full name of Mr. Cheng Wang (Tongren University), one of the collectors of the type specimens; noun (name) in genitive case.

#### Diagnosis.

**Figure 9.**
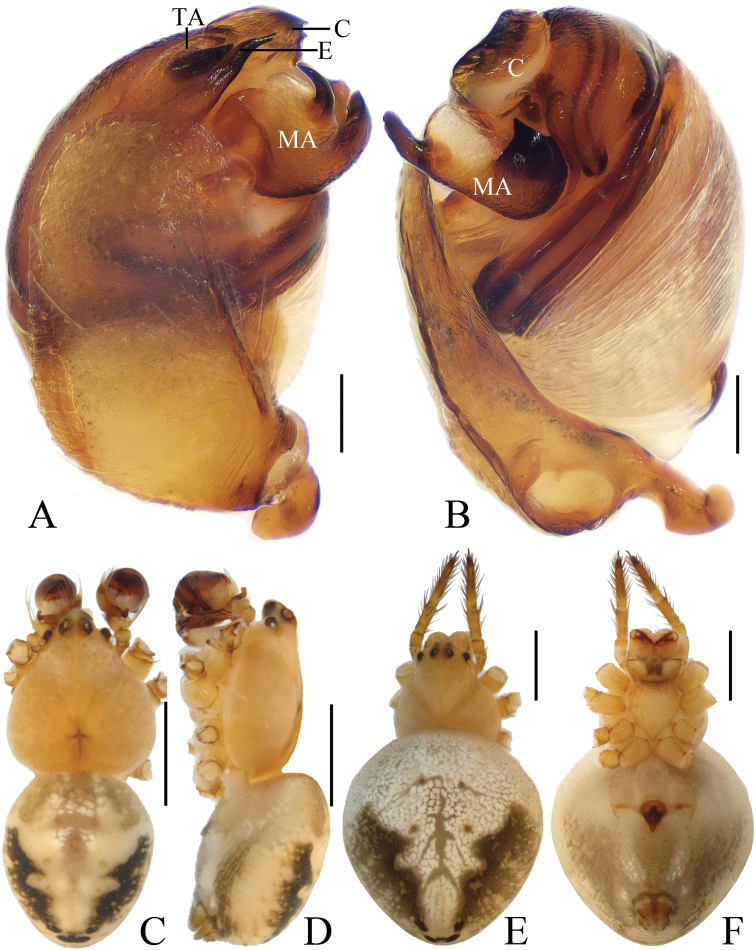
*Eriovixia yaoi* sp. nov., male holotype and female paratype **A** male palp, dorsal view **B** ibid., prolateral view **C** holotype habitus, dorsal view **D** ibid., lateral view **E** paratype habitus, dorsal view **F** ibid., ventral view. Scale bars: 0.1 mm (**A, B**); 1 mm (**C–F**).

**Figure 10.**
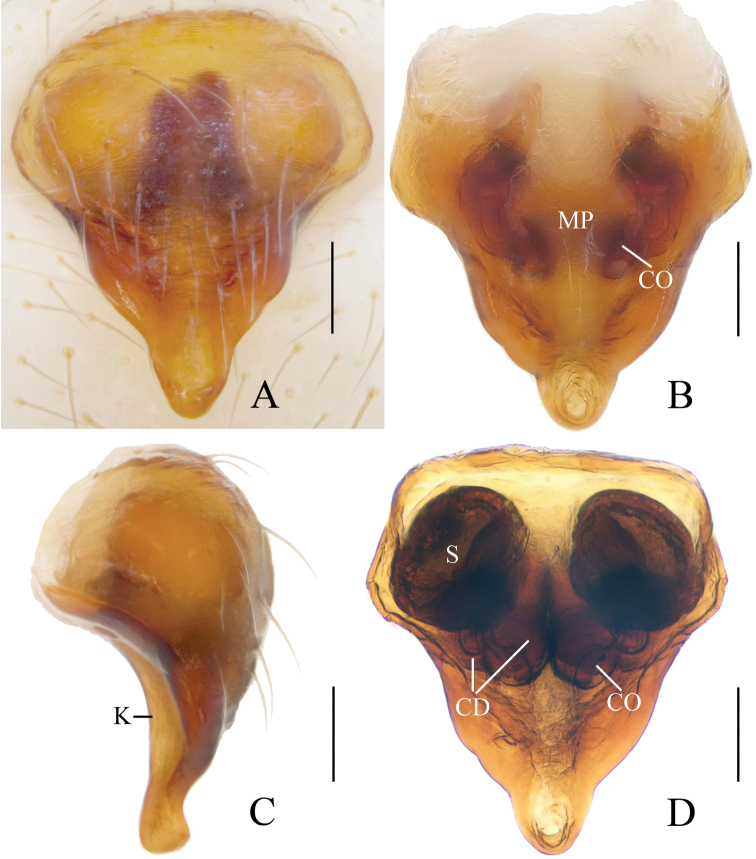
*Eriovixia yaoi* sp. nov., female paratype, epigyne **A** ventral view **B** dorsal view **C** lateral view **D** dorsal view. Scale bars: 0.1 mm.

*Eriovixia wangchengi* sp. nov. resembles *E. yaoi* sp. nov. and also *E. jianfengensis* by habitus and copulatory organs, but can be distinguished from them by: 1) posterior end of the abdomen with 6 tubercles vs. lacking (Figs 9C–F; Han and Zhu 2010: figs 2B–D); 2) PMEs of male anterior to AMEs in lateral view vs. AMEs anterior to PMEs (Figs 19E, 21D; Han and Zhu 2010: fig. 2D); 3) conductor with a tapered tip, visible in apical view vs. no tapered tip; 4) embolus straight in prolateral view vs. curved (Fig. 9A; Han and Zhu 2010: fig. 9D); 5) spermathecae touching each other vs. separated (Fig. 10D; Han and Zhu 2010: fig. 9A); and 6) scape not keeled vs. keeled (Fig. 10C, Han and Zhu 2010: fig. 9C). The male differs from *E. yaoi* also by having: 1) a cephalic protuberance vs. absent (Fig. 19E); and 2) long bristles around the eye region vs. absent (Figs 9C–D, 19E)

#### Description.

Male (holotype, Figs 7A–D, 17D, 19D, 20D). Total length 4.10. Carapace 2.10 long, 1.90 wide. Abdomen 2.10 long, 2.20 wide. Clypeus 0.18 high. Eye sizes and interdistances: AME 0.13, ALE 0.08, PME 0.13, PLE 0.08, AME–AME 0.15, AME–ALE 0.28, PME–PME 0.20, PME–PLE 0.40, MOA length 0.35 with anterior width 0.38 and posterior width 0.35. Leg measurements: I 8.70 (2.95, 3.15, 1.90, 0.70), II 5.65 (2.00, 1.95, 1.05, 0.65), III 3.60 (1.30, 1.15, 0.70, 0.45), IV 5.20 (1.70, 1.75, 1.20, 0.55). Carapace yellow, PME protruding over AME, with several long, dark bristles in median ocular area and behind lateral eyes, cervical groove inconspicuous. Chelicerae, endites, labium and sternum yellow. Legs yellow with inconspicuous rings. Abdomen about 1.05 times wider than long, covered with sparse, long setae, dorsum with dark markings anteriorly and a pair of arcuate stripes laterally, posteriorly with 6 tubercles; ventre greyish-brown with a pair of white spots anterior to the spinnerets.

*Palp* (Figs 7A, B, 17D): median apophysis with 2 branches; embolus straight, pointed apically; conductor with a tapered tip near the prolateral branch of the median apophysis in apical view; terminal apophysis about 1.8 times longer than wide in apical view, fused with embolus at base and bifurcated distally.

***Female*** (paratype IZCAS-Ar41678, Figs 7E, F, 8). Total length 4.70. Carapace 2.00 long, 1.80 wide. Abdomen 3.60 long, 3.50 wide. Clypeus 0.10 high. Eye sizes and interdistances: AME 0.10, ALE 0.08, PME 0.10, PLE 0.05, AME–AME 0.15, AME–ALE 0.23, PME–PME 0.18, PME–PLE 0.35, MOA length 0.33 with anterior width 0.33 and posterior width 0.33. Leg measurements: I 7.95 (2.75, 2.90, 1.60, 0.70), II 5.90 (1.90, 2.10, 1.25, 0.65), III 3.45 (1.15, 1.20, 0.60, 0.50), IV 5.20 (1.75, 1.80, 1.10, 0.55). Habitus similar to that of male, but abdomen dorsally with 2 pairs of lateral, arcuate stripes.

*Epigyne* (Fig. 8) pentagonal, about 1.3 times longer than wide, with a triangular scape; copulatory openings round; copulatory ducts directed ventrally at their origin, then turning dorsally, connected to the spermathecae at the posterior surface; spermathecae globular, touching each other.

#### Variation.

Total length: ♂♂ 3.90–4.60; ♀♀ 4.50–4.90.

#### Distribution.

China (Yunnan).

### Eriovixia yaoi sp. nov.

Taxon classificationAnimaliaAraneaeAraneidae

B4384E76-299E-54FA-9F24-6BD02C63DA10

http://zoobank.org/003C9A32-32DB-471A-822F-EE5B6B26F653

Figs 9 , 10 , 18A , 19E , 20E

#### Type material.

Holotype ♂ (IZCAS-Ar41684), China: Yunnan, Xishuangbanna, Mengla County, Menglun Township, Menglun Nature Reserve, Lvshilin Forest Park, limestone seasonal rainforest (21°54.61'N, 101°16.89'E, 640 m alt.), 14.XI.2009, G. Tang et al. leg. Paratypes: 1♂2♀ (IZCAS-Ar41685–41687), same data as holotype; 1♀(IZCAS-Ar41688), secondary seasonal moist forest (21°54.54'N, 101°17.20'E, 713 m alt.), 1–9.X.2006, G. Zheng leg.; 1♀ (IZCAS-Ar41689), same locality (21°54.54'N, 101°17.20'E, 713 m alt.), 19–25.X.2006, G. Zheng leg.; 1♂ (IZCAS-Ar41690), same locality (21°54.72'N, 101°16.94'E, 645 m alt.), 27.VII.2007 G. Zheng leg.; 1♂ (IZCAS-Ar41691), same locality (21°54.61'N, 101°17.01'E, 633 m alt.), 28.VII.2007 a.m. G. Zheng leg. Other material examined: 4♂2♀ (IZCAS-Ar41692), same locality (21°54.39'N, 101°16.81'E, 612 m alt.), 10.VIII.2007 a.m. G. Zheng leg.; 2♀ (IZCAS-Ar41693), secondary tropical montane evergreen broad-leaved forest (21°57.53'N, 101°12.38'E, 899 m alt.), 6.VIII.2007, G. Zheng leg.; 1♂ (IZCAS-Ar41694), primary tropical seasonal rainforest (21°57.67'N, 101°11.89'E, 790 m alt.), 7.VIII.2007 a.m. G. Zheng leg.; 1♂1♀ (IZCAS-Ar41695), *Anogeissus acuminata* plantation (about 20 yr) (21°53.99'N, 101°16.81'E, 611 m alt.), 19.VIII.2007 a.m. G. Zheng leg.; 1♀ (IZCAS-Ar41696), Lvshilin Forest Park (21°54.71'N, 101°16.90'E, 656 m alt.), 13.XI.2009, G. Tang et al. leg.; 1♂ (IZCAS-Ar41697), same locality (21°54.71'N, 101°16.90'E, 664 m alt.), 15.XI.2009, G. Tang et al. leg.; 2♂1♀ (IZCAS-Ar41698), same locality (21°54.71'N, 101°16.94'E, 652 m alt.), 16.XI.2009, G. Tang et al. leg.; 1♀ (IZCAS-Ar41699), same locality (21°54.71'N, 101°16.94'E, 660 m alt.), 16.XI.2009, G. Tang et al. leg.; 1♀ (IZCAS-Ar41700), same locality (21°54.56'N, 101°16.86'E, 615 m alt.), 29.XI.2009, G. Tang et al. leg.; 1♂ (IZCAS-Ar41701), same locality (21°54.68'N, 101°16.95'E, 637 m alt.), 10.VIII.2018 night, C. Wang et al. leg; 2♀ (IZCAS-Ar41702), same locality (21°54.38'N, 101°16.82'E, 627 m alt.), 23.XI.2009, G. Tang et al. leg.;1♂ (IZCAS-Ar41703), same locality (21°54.58'N, 101°16.50'E, 566.2 m alt.), 28.IV.2019 night, C. Wang et al. leg; 1♀ (IZCAS-Ar41704), same locality (21°54.38'N, 101°16.82'E, 627 m alt.), 23.XI.2009, G. Tang et al. leg.; 1♂ (IZCAS-Ar41705), G213 roadside (21°54.12'N, 101°16.93'E, 590 m alt.), 24.XI.2009, G. Tang et al. leg.; 2♀ (IZCAS-Ar41706), same locality (21°54.09'E, 101°17.02'E, 570 m alt.), 28.XI.2009, G. Tang et al. leg.; 1♀ (IZCAS-Ar41707), G213 roadside (21°53.83'N, 101°17.00'E, 618 m alt.), 25.XI.2009, G. Tang et al. leg.; 1♀ (IZCAS-Ar41708), same locality (21°53.65'N, 101°16.98'E, 589 m alt.), 26.XI.2009, G. Tang et al. leg.; 1♂ (IZCAS-Ar41709), banyan plantation (21°54.09'E, 101°17.02'E, 579 m alt.), 28.XI.2009, G. Tang et al. leg.; 1♀ (IZCAS-Ar41710), G213 roadside (21°53.99'N, 101°16.95'E, 590 m alt.), 2.XII.2009, G. Tang et al. leg.; 1♀ (IZCAS-Ar41711), same locality (21°53.80'N, 101°17.08'E, 604.0 m alt.), 30.VII.2018, Z. Bai et al. leg; 2♀ (IZCAS-Ar41712), G213 roadside near Lvshilin (21°53.28'N, 101°16.75'E, 629.0 m alt.), 25.IV.2019, C. Wang et al. leg; 2♂ (IZCAS-Ar41713), G213 roadside (21°54.34'N, 101°16.79'E, 618 m alt.), 2.V.2019, Y. Tong et al.; 2♀ (IZCAS-Ar41714), #3 site in Mafengzhai Village (21°53.68'N, 101°17.33'E, 539 m alt.), 8.V.2019, Y. Tong et al.

#### Comparative material.

*Eriovixia jianfengensis* Han & Zhu, 2010, 1♂ (TRU), CHINA: Hainan, Ledong County, Jianfeng Township, Jianfengling National Natural Reserve (18°44.45'N, 108°51.49'E, 856 m alt.), 11.IV.2019, C. Wang et al. leg.; 2♀ (TRU), same locality (18°44.61'N, 108°51.24'E, 812 m alt.), 12.IV.2019, C. Wang et al. leg.

#### Etymology.

The specific name is a patronym of Dr. Zhiyuan Yao (College of Life Sciences, Shenyang Normal University), one of the collectors of the type specimens; noun (name) in genitive case.

#### Diagnosis.

*Eriovixia yaoi* sp. nov. resembles *E. jianfengensis* Han & Zhu, 2010 in habitus and copulatory organs, but differs in: 1) male lacking cephalic protuberance vs. present (Fig. 21D); 2) inner branch of the terminal apophysis longer than outer branch vs. inner branch shorter than outer branch (Fig. 21A); 3) narrower conductor vs. wider (Fig. 21A); 4) epigyne triangular and slightly lobed (enlarged medially) vs. triangular, without lobes (Han and Zhu 2010: figs 9A, B); and 5) carapace unicolour vs. darker in ocular region with dark, thin medial line on cephalic region (Han and Zhu 2010: figs 2B–D).

#### Description.

***Male*** (holotype, Figs 9A–D, 18A, 19E, 20E). Total length 3.30. Carapace 1.70 long, 1.40 wide. Abdomen 1.60 long, 1.40 wide. Clypeus 0.18 high. Eye sizes and interdistances: AME 0.10, ALE 0.08, PME 0.10, PLE 0.08, AME–AME 0.10, AME–ALE 0.15, PME–PME 0.15, PME–PLE 0.18, MOA length 0.30 with anterior width 0.33 and posterior width 0.30. Leg measurements: I 6.50 (2.10, 2.35, 1.50, 0.55), II 4.60 (1.50, 1.55, 1.05, 0.50), III 3.20 (1.05, 1.05, 0.65, 0.45), IV 4.50 (1.40, 1.55, 1.10, 0.45). Carapace yellowish-white, cervical groove obvious, cephalic protuberance lacking. Chelicerae, endites, labium and sternum yellow. Legs yellowish-brown, with dark rings on tibia of legs III-IV. Abdomen about 1.2 times longer than wide, dorsum with a large, pale folium, black-brown posterolaterally; ventre greyish-yellow with brown markings laterally.

*Palp* (Figs 9A, B, 18A): median apophysis with 2 pointed branches; embolus conical, longer than terminal apophysis in prolateral view, slightly curved near tip of terminal apophysis; terminal apophysis elliptical in apical view, bifurcated distally.

***Female*** (paratype IZCAS-Ar41685, Figs 9E, F, 10). Total length 4.40. Carapace 1.90 long, 1.50 wide. Abdomen 3.10 long, 2.90 wide. Clypeus 0.08 high. Eye sizes and interdistances: AME 0.13, ALE 0.08, PME 0.10, PLE 0.08, AME–AME 0.10, AME–ALE 0.18, PME–PME 0.15, PME–PLE 0.23, MOA length 0.30 with anterior width 0.33 and posterior width 0.33. Leg measurements: I 6.85 (2.25, 2.45, 1.50, 0.65), II 6.05 (1.95, 2.05, 1.45, 0.60), III 3.50 (1.15, 1.20, 0.70, 0.45), IV 5.15 (1.75, 1.80, 1.10, 0.50). Habitus similar to that of male, but cervical groove more conspicuous.

*Epigyne* (Fig. 10) about 1.2 times longer than wide, with a triangular, laterally constricted scape; median plate keeled; copulatory openings arcuate in posterior view; copulatory ducts directed ventrally and extended anteriorly, then ventrally again, connected to the spermathecae at its posterior surface; spermathecae globular, separated from each other.

#### Variation.

Total length: ♂♂ 3.30–3.40; ♀♀ 3.90–4.40.

#### Distribution.

China (Yunnan).

### Eriovixia yinae sp. nov.

Taxon classificationAnimaliaAraneaeAraneidae

4DBD503B-666D-5F0E-8AF6-1B3EE6AD8FC5

http://zoobank.org/7A53F81F-7301-4932-97ED-75BA28297B6A

**Figure 11.**
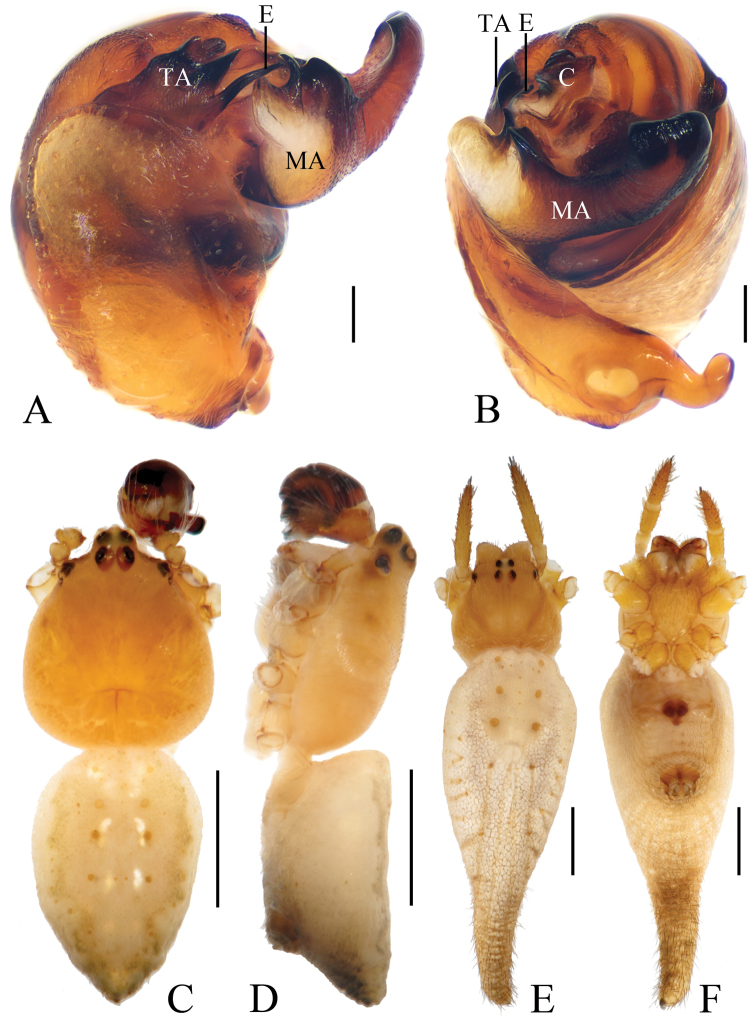
*Eriovixia yinae* sp. nov., male holotype and female paratype **A** male palp, dorsal view **B** ibid., prolateral view **C** holotype habitus, dorsal view **D** ibid., lateral view **E** paratype habitus, dorsal view **F** ibid., ventral view. Scale bars: 0.1 mm (**A, B**); 1 mm (**C–F**).

**Figure 12.**
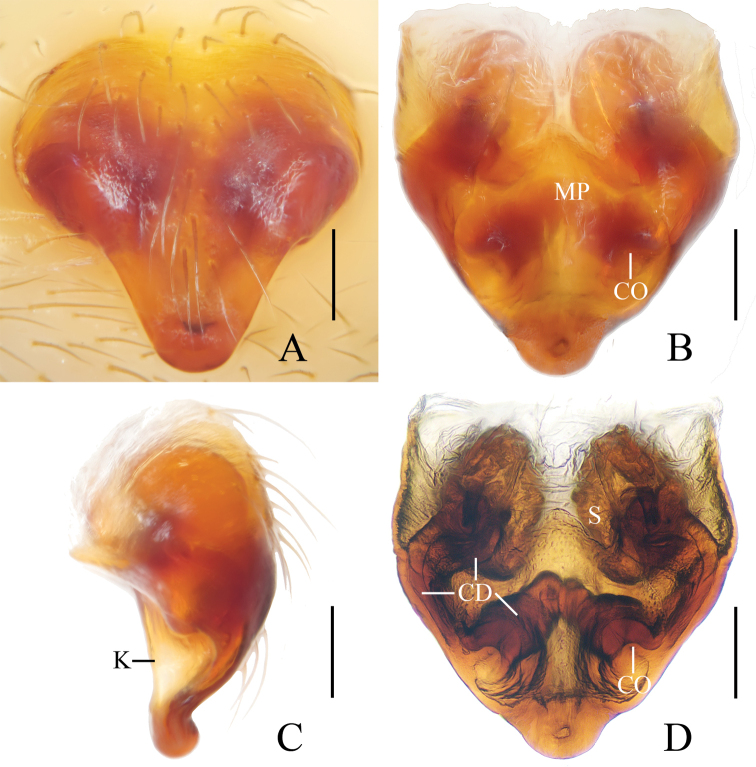
*Eriovixia yinae* sp. nov., female paratype, epigyne **A** ventral view **B** dorsal view **C** lateral view **D** dorsal view. Scale bars: 0.1 mm.

Figs 11 , 12 , 18B , 19F , 20F

#### Type material.

Holotype ♂ (IZCAS-Ar41715), CHINA: Yunnan, Xishuangbanna, Mengla County, Menglun Township, Menglun Nature Reserve, G213 roadside (21°53.65'N, 101°16.98'E, 589 m alt.), 26.XI.2009, G. Tang et al. leg. Paratypes: 3♂1♀ (IZCAS-Ar41716–41719), same data as holotype.

#### Etymology.

The specific name is a patronym in honour of the late Prof. Changmin Yin, one of the pioneers of spider taxonomy in China; noun (name) in genitive case.

#### Diagnosis.

*Eriovixia yinae* sp. nov. resembles *E. pseudocentrodes* (Bösenberg & Strand, 1906) by the copulatory organs, but can be distinguished from the latter by: 1) median plate keeled vs. not keeled (Mi et al. 2010: fig. 12); 2) spermathecae separated from each other vs. touching (Mi et al. 2010: figs 11 and 13); 3) posterior edge of PMEs and anterior edge of ALEs of male almost in a line in dorsal view vs. recurved (Mi et al. 2010: fig. 10); and 4) retrolateral branch of median apophysis with parallel margins in apical view vs. widest at distal end (Mi et al. 2010: figs 14–15).

#### Description.

***Male*** (holotype, Figs 11A–D, 18B, 19F, 20F). Total length 3.50. Carapace 1.70 long, 1.40 wide. Abdomen 1.90 long, 1.30 wide. Clypeus 0.13 high. Eye sizes and interdistances: AME 0.10, ALE 0.08, PME 0.10, PLE 0.08, AME–AME 0.13, AME–ALE 0.25, PME–PME 0.10, PME–PLE 0.30, MOA length 0.28 with anterior width 0.30 and posterior width 0.28. Leg measurements: I 5.85 (1.80, 2.15, 1.35, 0.55), II 4.80 (1.50, 1.70, 1.10, 0.50), III 2.75 (0.85, 1.00, 0.55, 0.35), IV 4.10 (1.25, 1.45, 1.00, 0.40). Carapace yellow, cervical groove inconspicuous, cephalic protuberance short, narrower than AME diameter. Chelicerae yellow with 4 promarginal teeth and 4 retromarginal teeth. Endites, labium and sternum coloured as chelicerae. Sternum with dark setae. Legs yellow, without rings. Abdomen about 1.5 times longer than wide, covered with long setae anteriorly, dorsum greyish with dark bordered folium, medially with 4 pairs of white spots; ventre greyish.

*Palp* (Figs 11A, B, 18B): median apophysis with lamella and 2 spurs prolaterally, retrolaterally slightly curled; embolus thin, slightly curved, longer than terminal apophysis in prolateral view; conductor curved to a tapered tip, guiding the embolus; terminal apophysis trapezoidal with translucent lamella in prolateral view, fused with embolus at base.

***Female*** (paratype IZCAS-Ar41716, Figs 11E, F, 12). Total length 6.60. Carapace 2.00 long, 1.70 wide. Abdomen 5.40 long, 2.10 wide. Clypeus 0.08 high. Eye sizes and interdistances: AME 0.10, ALE 0.08, PME 0.10, PLE 0.05, AME–AME 0.13, AME–ALE 0.20, PME–PME 0.15, PME–PLE 0.25, MOA length 0.28 with anterior width 0.28 and posterior width 0.30. Leg measurements: I 7.10 (2.25, 2.70, 1.55, 0.60), II 5.80 (1.75, 2.20, 1.30, 0.55), III 3.20 (1.00, 1.15, 0.65, 0.40), IV 4.90 (1.50, 1.75, 1.15, 0.50). Habitus similar to that of male, but abdomen about 2.6 times longer than wide.

*Epigyne* (Fig. 12) triangular, about 1.1 times wider than long; median plate strongly keeled; copulatory openings round; copulatory ducts long, directed medially from their origin, then turned to the lateral edge and twisted to connect to the spermathecae ventrally; spermathecae ovoid, separated from each other.

#### Variation.

Total length: ♂♂ 3.50–4.20.

#### Distribution.

China (Yunnan).

### Eriovixia yunnanensis

Taxon classificationAnimaliaAraneaeAraneidae

(Yin, Wang, Xie & Peng, 1990)

A5712D16-88B2-5E09-B1EC-C70887C7029D

**Figure 13.**
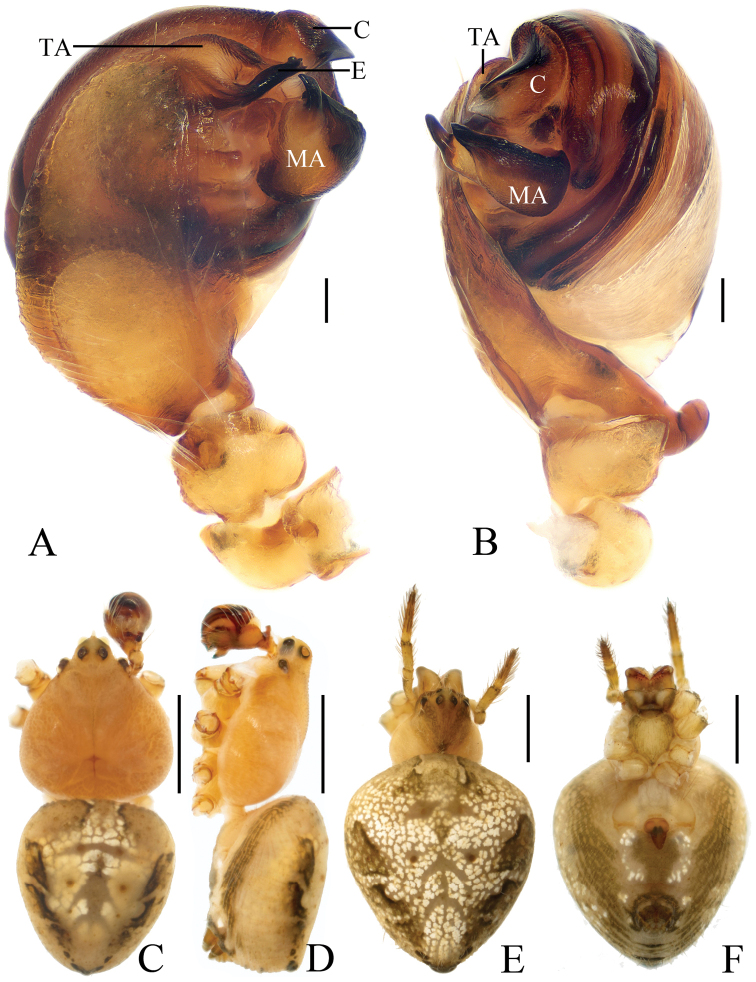
*Eriovixia yunnanensis***A** male palp, dorsal view **B** ibid., prolateral view **C** male habitus, dorsal view **D** ibid., lateral view **E** female habitus, dorsal view **F** ibid., ventral view. Scale bars: 0.1 mm (**A, B**); 1 mm (**C–F**).

**Figure 14.**
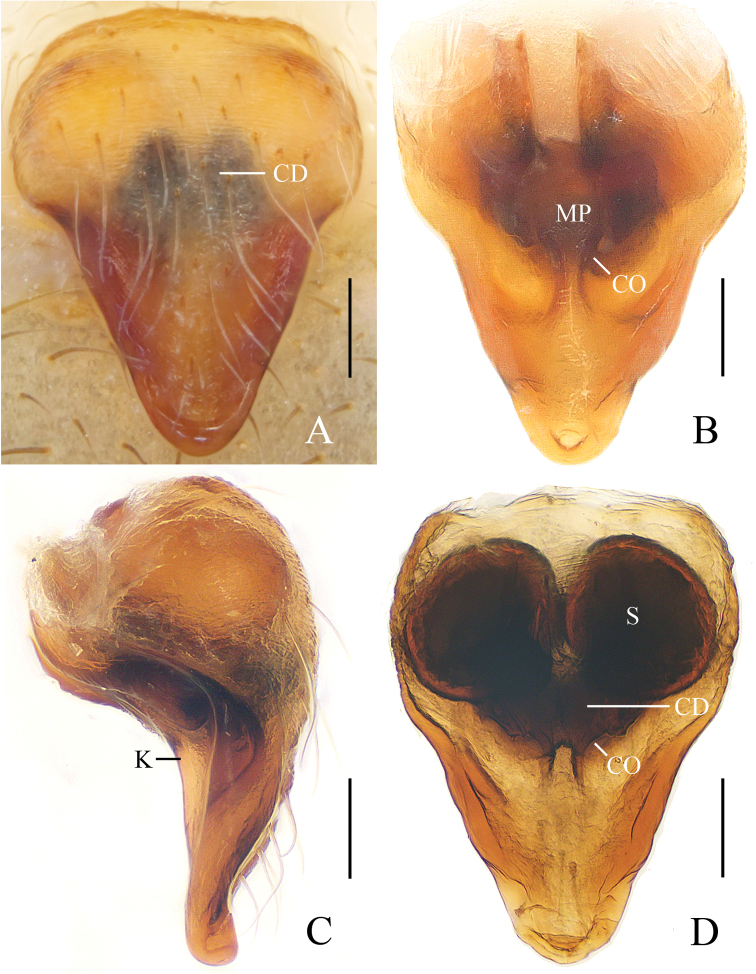
*Eriovixia yunnanensis*, epigyne **A** ventral view **B** dorsal view **C** lateral view **D** dorsal view. Scale bars: 0.1 mm.

Figs 13 , 14 , 18C , 19G , 20G

Neoscona yunnanensis Yin et al., 1990: 115, figs 283–287 (♀).
Eriovixia yunnanensis : Yin et al., 1997: 302, figs 209a–g (♀); Song, Zhu and Chen, 1999: 281, figs 167B, C and M (♀).

#### Type material examined.

Holotype ♀ (HNU), China: Yunnan, 2.VIII.1981, J.F. Wang leg.

#### Other material examined.

1♀ (IZCAS-Ar41720), China: Yunnan, Xishuangbanna, Mengla County, Menglun Township, Menglun Nature Reserve, secondary tropical seasonal rainforest (21°55.43'N, 101°16.44'E, 598 m alt.), 16–24.VIII.2006, G. Zheng leg.; 1♀ (IZCAS-Ar41721), primary tropical seasonal rainforest (21°55.04'N, 101°16.50'E, 558 m alt.), 22.VII.2007 p.m., G. Zheng leg.; 1♂ (IZCAS-Ar41722), same locality (21°57.59'N, 101°12.21'E, 822 m alt.), 8.VIII.2007 a.m. G. Zheng leg.; 1♂ (IZCAS-Ar41723), *Magnolia baillonii* plantation (about 20 yr) (21°54.20'N, 101°16.92'E, 608 m alt.), 18.VIII.2007 a.m. G. Zheng leg.; 1♀ (IZCAS-Ar41724), *Anogeissus acuminata* plantation (about 20 yr) (21°53.99'N, 101°16.81'E, 611 m alt.), 19.VIII.2007 a.m. G. Zheng leg.; 1♂ (IZCAS-Ar41725), Lvshilin Forest Park (21°54.71'N, 101°16.90'E, 664 m alt.), 15.XI.2009, G. Tang et al. leg.; 2♀ (IZCAS-Ar41726), G213 roadside (21°54.46'N, 101°16.76'E, 644 m alt.), 20.XI.2009, G. Tang et al. leg.; 1♂ (IZCAS-Ar41727), G213 roadside (21°54.38'N, 101°16.82'E, 620 m alt.), 21.XI.2009, G. Tang et al. leg.; 1♂ (IZCAS-Ar41728), same locality (21°54.39'N, 101°16.80'E, 627 m alt.), 22.XI.2009, G. Tang et al. leg.; 2♂ (IZCAS-Ar41729), secondary tropical forest near Lvshilin Forest Park (21°54.38'N, 101°16.82'E, 627 m alt.), 23.XI.2009, G. Tang et al. leg.; 1♀ (IZCAS-Ar41730), same locality (21°54.38'N, 101°16.82'E, 627 m alt.), 23.XI.2009, G. Tang et al. leg.; 1♂2♀ (IZCAS-Ar41731–41733), G213 roadside (21°53.67'N, 101°16.98'E, 589 m alt.), 26.XI.2009, G. Tang et al. leg.; 1♂1♀ (IZCAS-Ar41734), same locality (21°53.62'N, 101°16.96'E, 581 m alt.), 26.XI.2009, G. Tang et al. leg.; 1♀ (IZCAS-Ar41735), G213 roadside (21°54.09'E, 101°17.02'E, 570 m alt.), 28.XI.2009, G. Tang et al. leg.; 1♂1♀ (IZCAS-Ar41736), Lvshilin Forest Park, limestone seasonal rainforest (21°54.56'N, 101°16.86'E, 610 m alt.), 29.XI.2009, G. Tang et al. leg.; 1♂ (IZCAS-Ar41737), tropical evergreen rainforest (21°55.14'N, 101°16.30'E, 523 m alt.), 30.XI.2009, G. Tang et al. leg.; 1♀ (IZCAS-Ar41738), valley tropical seasonal rainforest (21°54.97'N, 101°16.43'E, 551 m alt.), 1.XII.2009, G. Tang et al. leg.; 1♂ (IZCAS-Ar41739), same locality (21°54.85'N, 101°16.55'E, 569 m alt.), 1.XII.2009, G. Tang et al. leg.; 1♂ (IZCAS-Ar41740), G213 roadside (21°53.99'N, 101°16.95'E, 590 m alt.), 2.XII.2009, G. Tang et al. leg.; 3♂2♀ (IZCAS-Ar41741), Yulinjiegou Scenic Spot (21°55.05'N, 101°16.24'E, 572.8 m alt.), 19.VII.2018, X. Mi et al. leg.; 4♂ (IZCAS-Ar41742), same locality (21°55.60'N, 101°15.50'E, 572.8 m alt.), 24.VII.2018, X. Mi et al. leg.; 2♂5♀ (IZCAS-Ar41743), same locality (21°55.13'N, 101°16.08'E, 552.4 m alt.), 5.VIII.2018, C. Wang et al. leg.; 1♂ (IZCAS-Ar41744), same locality (21°55.40'N, 101°16.36'E, 584 m alt.), 11.VIII.2018, C. Wang et al. leg.; 3♂6♀ (IZCAS-Ar41745), G213 roadside (21°52.65'N, 101°16.27'E, 575.0 m alt.), 31.VII.2018, Z. Bai et al. leg.; 2♀ (IZCAS-Ar41746), Masuoxing Village (21°54.02'N, 101°16.90'E, 561 m alt.), 27.IV.2019, Y. Tong et al. leg.; 1♀ (IZCAS-Ar41747), G213 roadside (21°54.34'N, 101°16.79'E, 618 m alt.), 2.V.2019, Y. Tong et al. leg.; 3♂ (IZCAS-Ar41748), #3 site in Mafengzhai Village (21°53.68'N, 101°17.33'E, 539 m alt.), 8.V.2019, Y. Tong et al. leg.

#### Comparative material.

*Eriovixia jianfengensis* Han & Zhu, 2010, 1♂, CHINA: Hainan, Ledong County, Jianfeng Township, Jianfengling National Natural Reserve (18°44.45'N, 108°51.49'E, 856 m alt.), 11.IV.2019 , C. Wang et al. leg.; 2♀, same locality (18°44.61'N, 108°51.24'E, 812 m alt.), 12.IV.2019, C. Wang et al. leg.

#### Diagnosis.

*Eriovixia yunnanensis* resembles *E. jianfengensis* in general appearance, but can be distinguished from the latter by: 1) branches of the median apophysis very close to each other vs. more than 90° apart (Han and Zhu 2010: figs 9D–E; Figs 21A–C); 2) tip of the embolus with parallel margins in prolateral view vs. tapered to a pointed tip (Han and Zhu 2010: fig. 9D; Figs 21A, C); and 3) spermathecae touching each other vs. separated (Han and Zhu 2010: figs 9A, B).

#### Description.

***Male*** (IZCAS-Ar41731, Figs 13A–D, 18C, 19G, 20G). Total length 3.40. Carapace 1.70 long, 1.60 wide. Abdomen 1.80 long, 1.60 wide. Clypeus 0.18 high. Eye sizes and interdistances: AME 0.10, ALE 0.08, PME 0.10, PLE 0.08, AME–AME 0.10, AME–ALE 0.18, PME–PME 0.13, PME–PLE 0.20, MOA length 0.30 with anterior width 0.33 and posterior width 0.30. Leg measurements: I 7.20 (2.35, 2.65, 1.55, 0.65), II 5.20 (1.75, 1.75, 1.20, 0.50), III 3.10 (1.05, 1.05, 0.60, 0.40), IV 4.55 (1.45, 1.55, 1.05, 0.50). Carapace yellowish-brown, cervical groove inconspicuous, cephalic protuberance narrower than AME diameter at base and about 1.5 AME diameter in length. Chelicerae, endites, labium and sternum yellow. Legs yellow, with inconspicuous dark rings. Abdomen about 1.1 times longer than wide, dorsum with a large, pale folium and with oblique dark markings posterolaterally; ventre greyish-brown with a pair of white arcuate patches and 2 pairs of white spots around the spinnerets.

*Palp* (Figs 13A, B, 18C): median apophysis bifurcated, 2 branches, close to each other; embolus curved, distal half with parallel margins in prolateral view; conductor curled distally; terminal apophysis as long as embolus, bifurcated distally.

***Female*** (IZCAS-Ar41732, Figs 13E, F, 14). Total length 4.10. Carapace 1.50 long, 1.40 wide. Abdomen 3.20 long, 2.70 wide. Clypeus 0.08 high. Eye sizes and interdistances: AME 0.10, ALE 0.08, PME 0.10, PLE 0.08, AME–AME 0.13, AME–ALE 0.15, PME–PME 0.10, PME–PLE 0.23, MOA length 0.28 with anterior width 0.30 and posterior width 0.30. Leg measurements: I 6.55 (2.20, 2.35, 1.40, 0.60), II 5.30 (1.75, 1.85, 1.15, 0.55), III 2.95 (1.00, 1.05, 0.50, 0.40), IV 4.50 (1.50, 1.55, 0.95, 0.50). Habitus similar to that of male, but cephalic region darker.

*Epigyne* (Fig. 14) triangular, about 1.25 times longer than wide; median plate keeled; copulatory openings arcuate; copulatory ducts short, extended anteriorly from their origin, then retraced and connected to the spermathecae at its posterior surface; spermathecae globular, touching each other.

#### Variation.

Total length: ♂♂ 3.20–3.60; ♀♀ 3.70–5.00.

#### Distribution.

China (Yunnan).

#### Comments.

The females collected from Xishuangbanna are almost identical to the holotype of *E. yunnanensis*.

### Eriovixia pengi sp. nov.

Taxon classificationAnimaliaAraneaeAraneidae

73DC884A-3F36-557F-B851-0464D3ACD793

http://zoobank.org/D45BC866-9125-41ED-A087-35B39EC54948

Eriovixia yunnanensis : Mi et al., 2010: 47, figs 25–33(♂♀, misidentification).

#### Type material.

Holotype ♂, China: Yunnan Province, Tengchong County, Jietou Township, Shaba Village (25°23.56'N, 98°42.21'E, 1850 m alt.), 25.V.2006, X.P. Wang et al. leg. (HNU-WANG060525). Paratypes: 1♀, Tengchong County, Jietou Township, Zhoujiapo Village (25°33.51'N, 98°39.97'E, 1660 m alt.), 16.V.2006, C.M. Yin et al. leg. (CAS-YHY03); 1♀, Tengchong County, Shangyin Township, Cuanlong Village (25°0.40'N, 98°42.60'E, 1990 m alt.), 4.VI.2006, C.M. Yin et al. leg. (HNU-YHY25); 1 ♂, Tengchong County, Jietou Township, Datang Village Daheling Ganjiao (25°25.21'N, 98°24.57'E, 1878 m alt.), 19.V.2006, X.J. Peng et al. leg. (HNU-PWH060519); 1 ♂, same data as HNU-PWH060519 (CAS-PWH060519).

#### Etymology.

The specific name is a patronym in honour of Prof. Xianjin Peng (College of Life Sciences, Hunan Normal University), one of the leading spider taxonomists of China; noun (name) in genitive case.

#### Diagnosis.

This new species is very similar to *E. yunnanensis* in appearance, but can be distinguished from the latter by: 1) having a thread-like terminal apophysis vs. not thread-like (Figs 13A, B and 18C); 2) the straight and pointed embolus vs. curved and non-tapering embolus (Fig. 13A); 3) the branches of the median apophysis about 45° apart vs. very close to each other (Figs 13A, B, 18C); 4) a pair of rhomboidal copulatory openings vs. arcuate (Fig. 14B, D). It is somewhat similar to *E. yaoi* sp. nov. by the copulatory organs and the habitus, but differs in having: 1) a thread-like terminal apophysis vs. bifurcated (Figs 9A, B, 18A); 2) straight embolus vs. curved (Fig. 9A); and 3) a pair of rhomboidal copulatory openings vs. arcuate (Figs 10B, D).

#### Description.

See Mi et al. (2010).

#### Distribution.

China (Yunnan).

### Eriovixia zhengi sp. nov.

Taxon classificationAnimaliaAraneaeAraneidae

4E5F437B-A3D9-56A3-A306-3443C18129D0

http://zoobank.org/9880E33C-46C1-43C1-8A57-FFF5F54684B9

**Figure 15.**
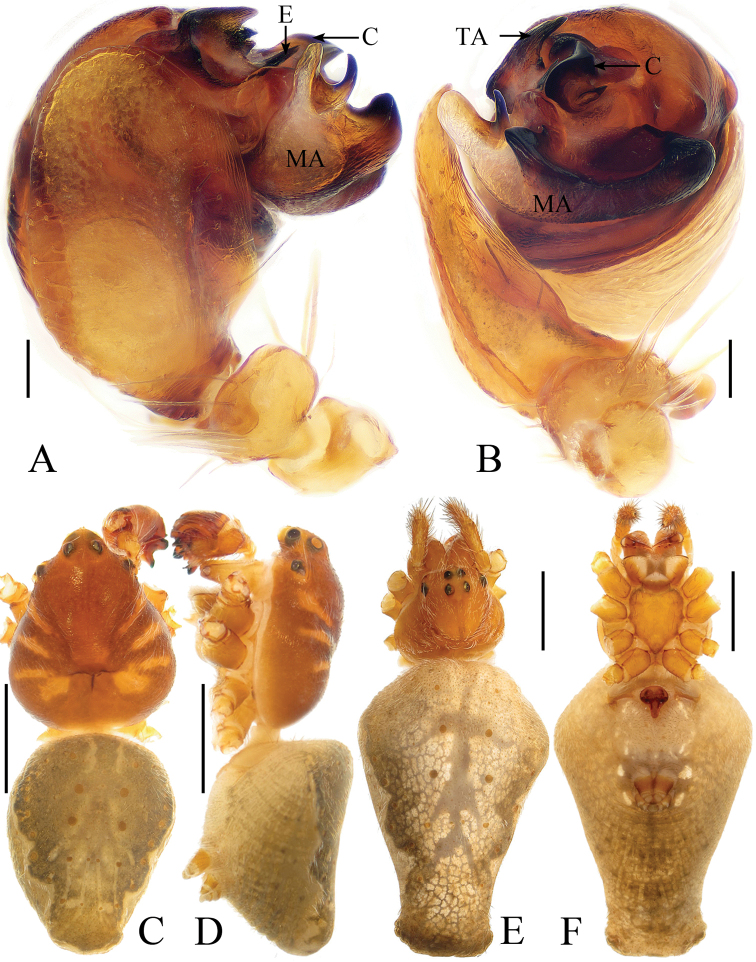
*Eriovixia zhengi* sp. nov., male holotype and female paratype **A** male palp, dorsal view **B** ibid., prolateral view **C** holotype habitus, dorsal view **D** ibid., lateral view **E** paratype habitus, dorsal view **F** ibid., ventral view. Scale bars: 0.1 mm (**A, B**); 1 mm (**C–F**).

**Figure 16.**
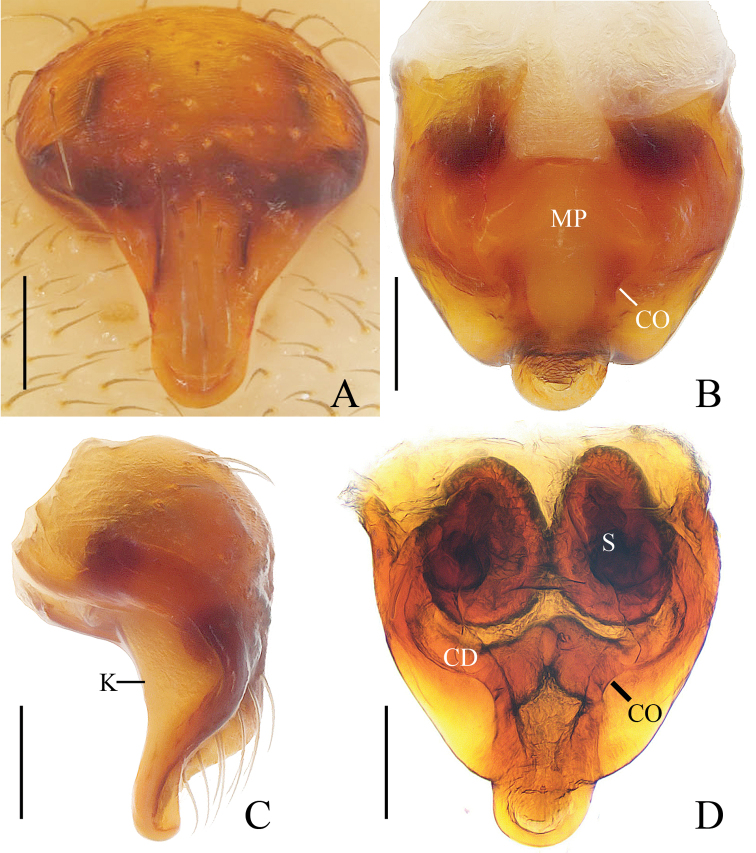
*Eriovixia zhengi* sp. nov., female paratype, epigyne **A** ventral view **B** dorsal view **C** lateral view **D** dorsal view. Scale bars: 0.1 mm.

Figs 15 , 16 , 18D , 19H , 20H

#### Type material.

Holotype ♂ (IZCAS-Ar41749), China: Yunnan, Xishuangbanna, Mengla County, Menglun Township, Menglun Nature Reserve, G213 roadside (21°54.12'N, 101°16.93'E, 590 m alt.), 24.XI.2009, G. Tang et al. leg. Paratypes: 2♀ (IZCAS-Ar41750–41751), same data as holotype; 1♀ (IZCAS-Ar41752), valley tropical seasonal rainforest (21°54.85'N, 101°16.55'E, 569 m alt.), 1.XII.2009, G. Tang et al. leg.; 1♂1♀ (IZCAS-Ar41753–41754), secondary tropical seasonal moist forest (21°54.72'N, 101°16.94'E, 645 m alt.), 27.VII.2007, G. Zheng leg.; 2♂ (IZCAS-Ar41755–41756), same locality (21°54.61'N, 101°17.01'E, 633 m alt.), 28.VII.2007 a.m. G. Zheng leg.; 1♀ (IZCAS-Ar41757), *Magnolia baillonii* plantation (about 20 yr) (21°54.20'N, 101°16.92'E, 608 m alt.), 18.VIII.2007 a.m. G. Zheng leg.; 1♂ (IZCAS-Ar41758), rubber plantation (21°54.70'N, 101°16.39'E, 593 m alt.), 12.XI.2009, G. Tang et al.; Other material examined: 1♂ (IZCAS-Ar41759), Lvshilin Forest Park, limestone seasonal rainforest (21°54.71'N, 101°16.94'E, 652 m alt.), 15.XI.2009, G. Tang et al. leg.; 1♂ (IZCAS-Ar41760), same locality (21°54.71'N, 101°16.94'E, 660 m alt.), 16.XI.2009, G. Tang et al. 2♂ (IZCAS-Ar41761), same locality (21°54.60'N, 101°17.08'E, 640 m alt.), 17.XI.2009, G. Tang et al. leg.; 1♂ (IZCAS-Ar41762), same locality (21°54.61'N, 101°17.09'E, 643 m alt.), 17.XI.2009, G. Tang et al. leg.; 1♂ (IZCAS-Ar41763), same locality (21°54.56'N, 101°16.86'E, 610 m alt.), 29.XI.2009, G. Tang et al. leg.; 1♂ (IZCAS-Ar41764), same locality (21°54.67'N, 101°16.98'E, 630 m alt.), 22.VII.2018 night, X. Mi et al. leg; 1♂ (IZCAS-Ar41765), same locality (21°54.67'N, 101°16.98'E, 630.1 m alt.), 30.VII.2018, X. Mi et al. leg; 1♀ (IZCAS-Ar41766), same locality (21°54.58'N, 101°16.50'E, 566.2 m alt.), 6.VIII.2018, C. Wang et al. leg; 2♂2♀ (IZCAS-Ar41767), same locality (21°54.65'N, 101°17.02'E, 686.3 m alt.), 9.VIII.2018, C. Wang et al. leg; 1♂ (IZCAS-Ar41768), same locality (21°54.68'N, 101°16.95'E, 637.1 m alt.), 10.VIII.2018 night, C. Wang et al. leg; 1♀ (IZCAS-Ar41769), G213 roadside (21°54.09'N, 101°17.02'E, 570 m alt.), 28.XI.2009, G. Tang et al. leg.; 1♂ (IZCAS-Ar41770), G213 roadside (21°54.09'N, 101°17.02'E, 579 m alt.), 28.XI.2009, G. Tang et al. leg.; 1♂ (IZCAS-Ar41771), G213 roadside (21°55.18'N, 101°16.37'E, 581 m alt.), 30.XI.2009, G. Tang et al. leg.; 1♀ (IZCAS-Ar41772), same locality (21°53.99'N, 101°16.95'E, 596 m alt.), 2.XII.2009, G. Tang et al. leg.; 2♂1♀ (IZCAS-Ar41773), same locality (21°54.02'N, 101°16.93'E, 606 m alt.), 2.VIII.2018, Z. Bai et al. leg; 3♀ (IZCAS-Ar41774), same locality (21°53.80'N, 101°17.08'E, 604 m alt.), 30.VII.2018, Z. Bai et al. leg; 1♀ (IZCAS-Ar41775), Xishuangbanna Tropical Botanical Garden, eastern part (21°54.07'N, 101°16.36'E, 544 m alt.), 22.VII.2018, X. Mi et al. leg; 2♂ (IZCAS-Ar41776), same locality (21°54.02'N, 101°16.90'E, 561 m alt.), 27.IV.2019, Y. Tong et al.; 1♂ (IZCAS-Ar41777), same locality, (21°55.065'N, 101°16.362'E, 579 m alt.), 10.VIII.2018, C. Wang et al. leg. 2♂1♀ (IZCAS-Ar41778), Mafengzhai Village (21°53.59'N, 101°17.33'E, 559 m alt.), 5.VIII.2018, Z. Bai et al. leg; 1♂ (IZCAS-Ar41779), same locality (21°53.45'N, 101°17.40'E, 543 m alt.), 29.IV.2019, Y. Tong et al. leg.; 1♂ (IZCAS-Ar41780), tropical rainforest (21°55.18'N, 101°16.06'E, 550 m alt.), 8.VIII.2018, C. Wang et al. leg.

#### Etymology.

The specific name is a patronym of Dr. Guo Zheng (College of Life Sciences, Shenyang Normal University), one of the collectors of the type specimens; noun (name) in genitive case.

#### Diagnosis.

*Eriovixia zhengi* sp. nov. resembles *E. pseudocentrodes*, *E. huwena* and *E. sticta* in appearance. It differs from *E. pseudocentrodes* by: 1) embolus fused with terminal apophysis at base in prolateral view vs. not fused (Mi et al. 2010: fig. 14); 2) conductor not curled to a pointed tip vs. curled to a pointed tip (Mi et al. 2010: Figs 14–15); 3) epigyne widest at base vs. widest medially (Mi et al. 2010: figs 11 and 13); 4) median plate keeled vs. not keeled (Mi et al. 2010: fig. 12); and 5) posteriorly, abdomen square vs. pointed (Mi et al. 2010: figs 9–10). It differs from *E. huwena* in: 1) the conductor not curled to a pointed tip vs. curled to a pointed tip (Han and Zhu 2010: figs 8A and B); 2) having a spur at the base of the conductor vs. absent (Han and Zhu 2010: figs 8A and B); and 3) the spermathecae ovoid and touching each other vs. kidney-shaped and separated (Mi and Wang 2016: figs 5 and 6). It differs from *E. sticta* in: 1) the median apophysis having a lamella on the prolateral end vs. absent (Mi et al. 2010: figs 22–24); 2) having a spur at the base of the conductor vs. absent (Mi et al. 2010: figs 22 and 23); 3) the epigyne widest at base vs. widest medially (Mi et al. 2010: figs 19 and 21); and 4) the median plate keeled vs. not keeled (Mi et al. 2010: fig. 20).

#### Description.

***Male*** (holotype, Figs 15A–D, 18D, 19H, 20H). Total length 3.90. Carapace 1.80 long, 1.50 wide. Abdomen 1.90 long, 1.50 wide. Clypeus 0.10 high. Eye sizes and interdistances: AME 0.10, ALE 0.08, PME 0.10, PLE 0.05, AME–AME 0.10, AME–ALE 0.28, PME–PME 0.13, PME–PLE 0.35, MOA length 0.33 with anterior width 0.30 and posterior width 0.30. Leg measurements: I 6.05 (1.95, 2.25, 1.35, 0.50), II 4.90 (1.55, 1.80, 1.10, 0.45), III 2.85 (1.00, 0.95, 0.55, 0.35), IV 4.25 (1.40, 1.45, 1.00, 0.40). Carapace yellow-brown with yellow radial patches, cervical groove conspicuous, cephalic protuberance wider than AME diameter at base and less than an AME diameter in length. Chelicerae, endites, labium and sternum pale yellow. Legs yellow with inconspicuous dark rings. Abdomen about 1.5 times longer than wide, approximately straight posteriorly, dorsum with a wide, greyish-brown folium; ventre greyish-yellow.

*Palp* (Figs 15A, B, 18D): median apophysis with a lamella and 2 spurs; embolus straight, as long as terminal apophysis in apical view; conductor bent at middle part and with pointed spur at base; terminal apophysis about 2 times longer than wide, with a spur close to embolus in apical view.

***Female*** (paratype IZCAS-Ar41752, Figs 15 E, F, 16). Total length 5.00. Carapace 1.60 long, 1.40 wide. Abdomen 3.70 long, 2.40 wide. Clypeus 0.08 high. Eye sizes and interdistances: AME 0.10, ALE 0.08, PME 0.10, PLE 0.05, AME–AME 0.13, AME–ALE 0.25, PME–PME 0.18, PME–PLE 0.30, MOA length 0.30 with anterior width 0.28 and posterior width 0.30. Leg measurements: I 6.20 (2.10, 2.35, 1.25, 0.50), II 5.10 (1.60, 1.95, 1.10, 0.45), III 2.90 (1.00, 1.00, 0.55, 0.35), IV 4.50 (1.55, 1.55, 0.95, 0.45). Habitus similar to those of male, but paler and with white markings in ventre. All the specimens possess a straight posterior end of abdomen.

*Epigyne* (Fig. 16) about 1.1 times longer than wide; median plate keeled; copulatory openings narrow, curved; copulatory ducts long, directed medially, then turning laterally forming a semicircle and connected to the spermathecae ventrally; spermathecae ovoid, touching each other.

#### Variation.

Total length: ♂♂ 3.40–3.90; ♀♀ 3.50–5.80.

#### Distribution.

China (Yunnan).

**Figure 17.**
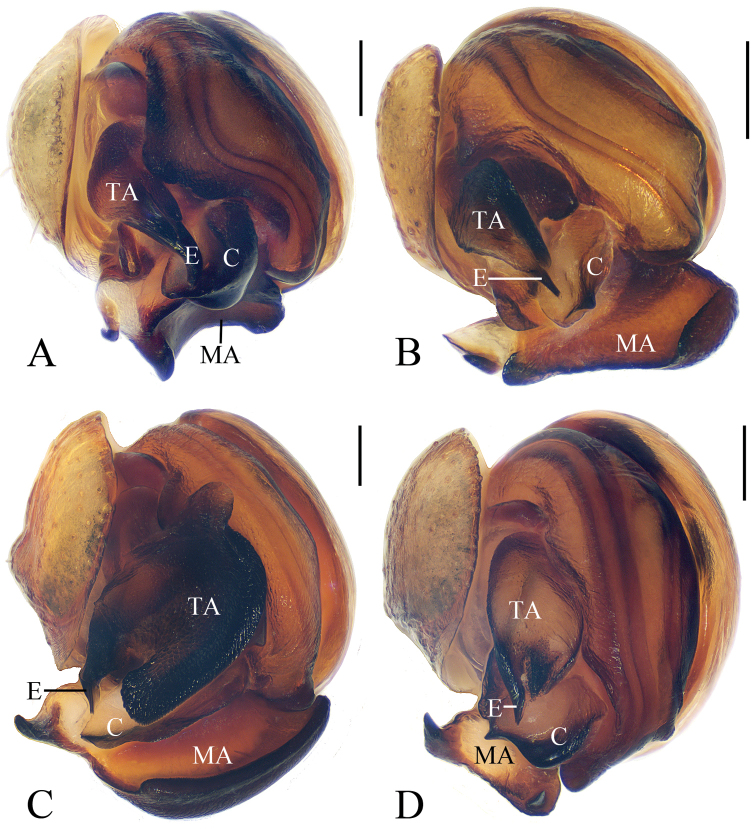
Male palps of *Eriovixia* spp, apical view **A***E. ganae* sp. nov. **B***E. liuhongi* sp. nov. **C***E. tangi* sp. nov. **D***E. wangchengi* sp. nov. Scale bars: 0.1 mm.

**Figure 18.**
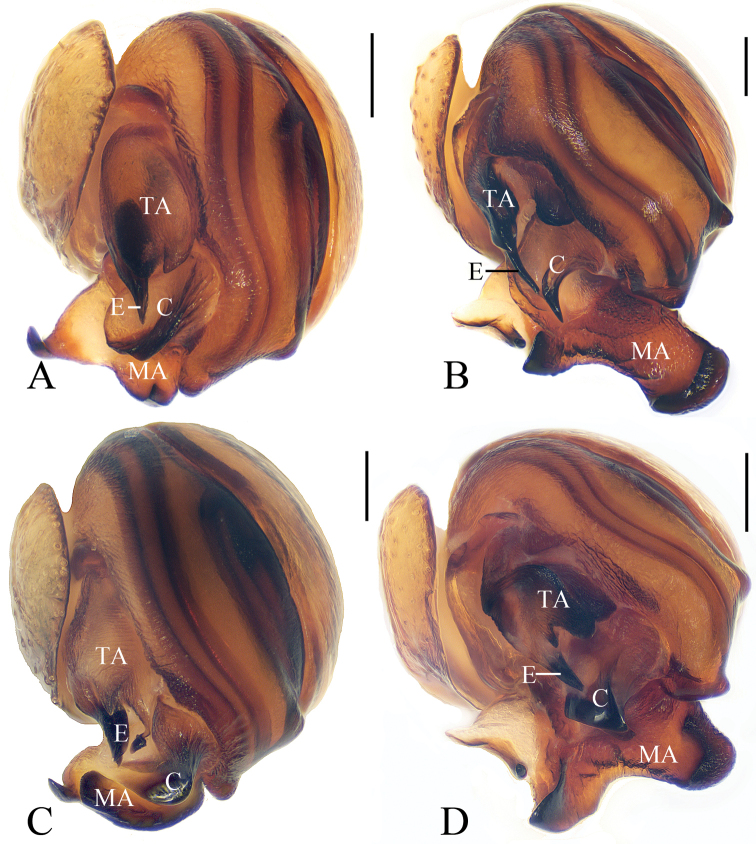
Male palps of *Eriovixia* spp, apical view **A***E. yaoi* sp. nov. **B***E. yinae* sp. nov. **C***E. yunnanensis***D***E. zhengi* sp. nov. Scale bars: 0.1 mm.

**Figure 19.**
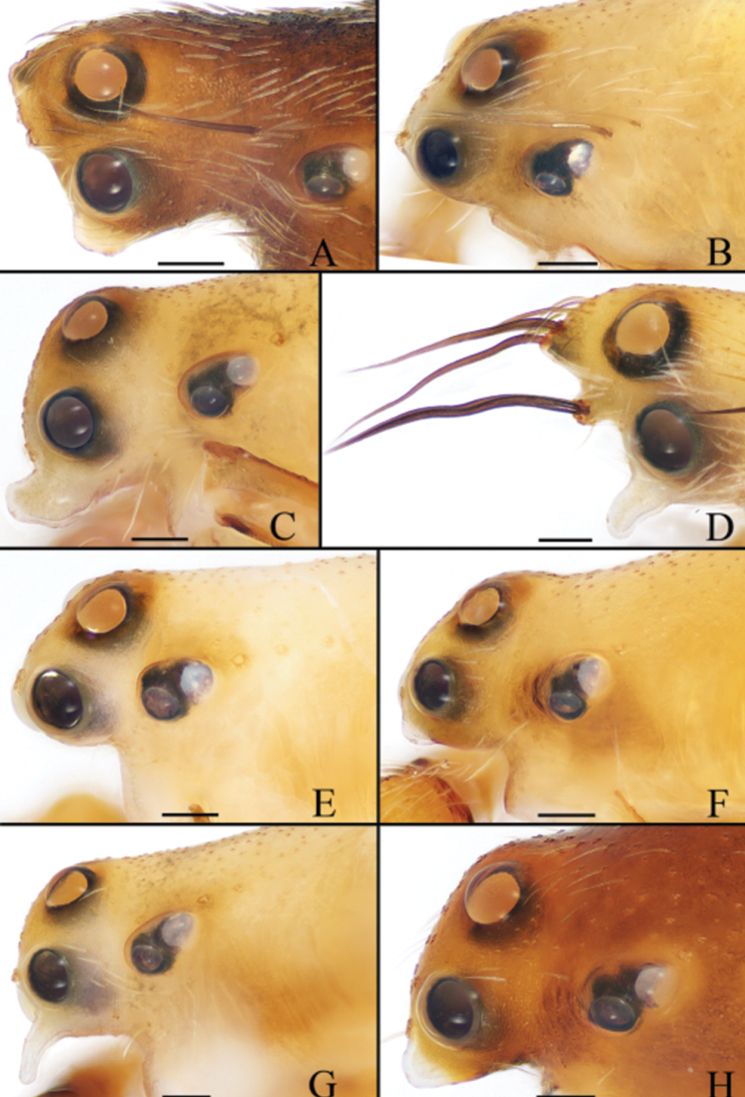
Eye regions of *Eriovixia* spp, lateral view **A***E. ganae* sp. nov. **B***E. liuhongi* sp. nov. **C***E. tangi* sp. nov. **D***E. wangchengi* sp. nov. **E***E. yaoi* sp. nov. **F***E. yinae* sp. nov. **G***E. yunnanensis***H***E. zhengi* sp. nov. Scale bars: 0.1 mm.

**Figure 20.**
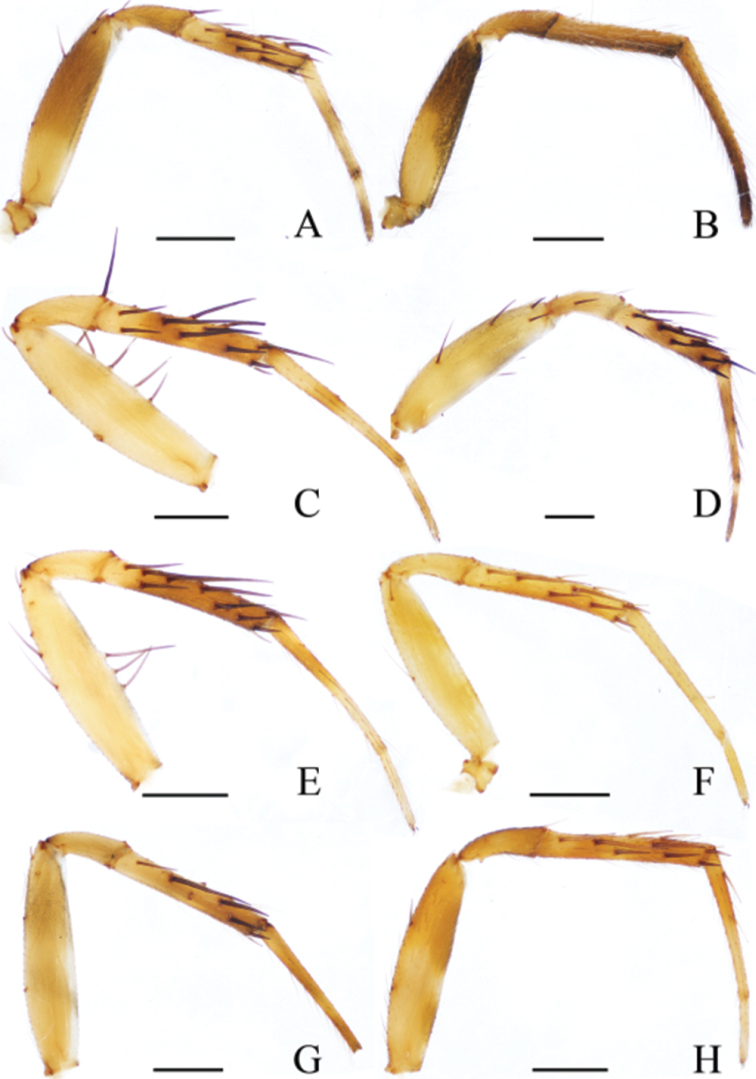
Leg II of *Eriovixia* spp, prolateral view **A***E. ganae* sp. nov. **B***E. liuhongi* sp. nov. **C***E. tangi* sp. nov. **D***E. wangchengi* sp. nov. **E***E. yaoi* sp. nov. **F***E. yinae* sp. nov. **G***E. yunnanensis***H***E. zhengi* sp. nov. **A, C–H** male **B** female. Scale bars: 0.5 mm.

**Figure 21.**
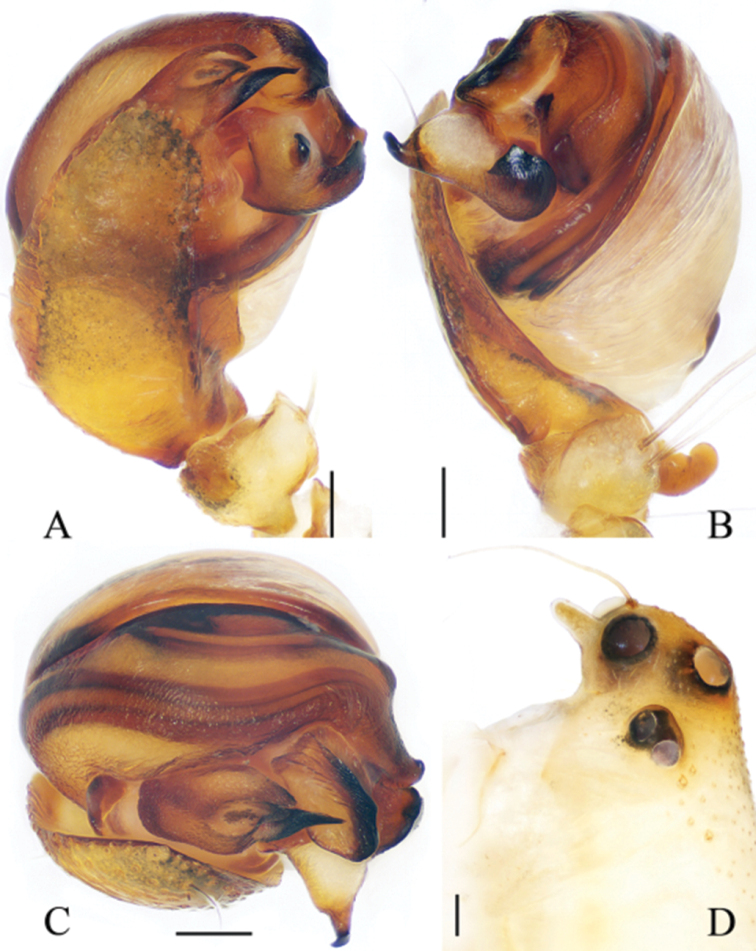
*Eriovixia jianfengensis* Han & Zhu, 2010 **A** male palp, dorsal view **B** ibid., prolateral view **C** ibid., apical view **D** eye region of male, lateral view. Scale bars: 0.1 mm.

## Supplementary Material

XML Treatment for Eriovixia

XML Treatment for Eriovixia ganae

XML Treatment for Eriovixia liuhongi

XML Treatment for Eriovixia tangi

XML Treatment for Eriovixia wangchengi

XML Treatment for Eriovixia yaoi

XML Treatment for Eriovixia yinae

XML Treatment for Eriovixia yunnanensis

XML Treatment for Eriovixia pengi

XML Treatment for Eriovixia zhengi
